# Long-term culturing of *Pseudomonas aeruginosa* in static, minimal nutrient medium results in increased pyocyanin production, reduced biofilm production, and loss of motility

**DOI:** 10.1128/aem.00975-25

**Published:** 2025-10-10

**Authors:** Rhiannon E. Cecil, Elana Ornelas, Anh Phan, Nahui Olin Medina-Chavez, Michael Travisano, Deborah R. Yoder-Himes

**Affiliations:** 1 Department of Biology, University of Louisville 5170, Louisville, Kentucky, USA; 2 Department of Ecology, Evolution and Behavior, University of Minnesota 172734 , St. Paul, Minnesota, USA; Centers for Disease Control and Prevention, Atlanta, Georgia, USA

**Keywords:** evolutionary biology

## Abstract

*Pseudomonas aeruginosa* is a multidrug-resistant opportunistic human pathogen that can survive in many natural and anthropogenic environments. It is a leading cause of morbidity in individuals with cystic fibrosis and is one of the most prevalent pathogens associated with nosocomial infections in the United States. It has been shown that this organism can survive and persist in low-nutrient environments, such as sink drains. How adaptation to these types of environments influences the phenotypic traits of this organism has not been well studied. Here, we implemented an experimental evolution system in which six strains of *P. aeruginosa* were subjected to low-nutrient conditions over the course of 12 weeks and assessed phenotypic and genotypic changes that occurred as a result of adaptation to such environments. We observed that adaptation to low-nutrient environments resulted in decreased generation time, reduced cell size, reduced biofilm formation, increased pyocyanin production, and decreased motility for some of the strains. Furthermore, some of the evolved isolates were significantly more virulent/competitive against a phagocytic predator. This study is significant as it allows us to predict how this organism will evolve in hospital and domestic environments and can help us improve treatment options for patients.

**IMPORTANCE:**

Human commensal and pathogenic organisms undergo dynamic cycles across human and non-human environments. Despite the crucial implications for human health, the understanding of bacterial adaptations to these diverse environments and their subsequent impact on human-bacterial interactions remains underexplored. This study shows how *Pseudomonas aeruginosa*, an opportunistic human pathogen, adapts phenotypically in response to a shift from high nutrients (like those found in the human body) to low nutrients (like those found in many other environments, like sink drains). This work also shows that, in some cases, resistance to predatory forces can evolve in the absence of a predator. This work is important as it contributes to the growing body of knowledge concerning how external, non-host-related abiotic conditions influence host–pathogen interactions.

## INTRODUCTION

Adaptations to environments with fluctuating nutrients, otherwise known as feast and famine cycles, are vitally important for microbial survival in various environmental niches, including natural environments, such as soil, aquatic, and the intestinal and respiratory tract of host organisms; as well as anthropogenic environments, such as drains and hospital equipment (1). Adaptations employed by some microorganisms in response to such environments, such as the stringent response (1–3), cellular dormancy via reduced cell growth and the formation of spores (4, 5), and metabolic shifting (fermentation vs respiration) (6, 7), are already well-studied. Our understanding of how these adaptations evolve in opportunistic human pathogens and the implications such adaptations have on virulence potential is relatively understudied. Uncovering the mechanisms behind these adaptations holds significant potential for ecological, evolutionary, environmental, industrial, and pharmaceutical applications. For example, these insights can be leveraged to better understand microbial diversity, microbial food webs, speciation, the evolution of bacterial communities, improve bioremediation techniques for cleaning polluted environments, develop more efficient industrial processes for biofuel production, and even design new antibiotics by targeting the pathways crucial for bacterial survival under nutrient limitations.

*Pseudomonas aeruginosa* is a member of the ESKAPE group of bacterial pathogens (8). It is an opportunistic pathogen and is known for its intrinsic multidrug resistance, genomic plasticity, ability to survive and form biofilms on abiotic surfaces, such as hospital equipment, and ability to evade the immune systems of humans, plants, and non-human animals (9–13). *P. aeruginosa* expresses with a wide arsenal of phenotypes that serve as a means of offensive and defensive protection against competitors and predators in the environment (e.g., protozoa, nematodes), including pyocyanin production, biofilm formation, and T3SS expression (14–16). Many of these phenotypes coincidentally act as virulence factors should the bacterium encounter a human host. *P. aeruginosa* is a well-studied organism, and how it adapts to host-associated niches, such as the nutrient-rich cystic fibrosis lung, has been researched in detail (17); however, how this organism evolves when forced to adapt to a low-nutrient, non-host-associated environment has not been thoroughly investigated. Furthermore, comparisons between adaptations to such an environment between *P. aeruginosa* isolates from clinical origin and those from an environmental origin remain under-explored, with many studies utilizing clinical isolates and excluding environmental isolates (18). However, a few studies have shown that clinical isolates may have larger or different accessory genomes compared to their environmental counterparts and more antibiotic resistance genes and mobile genetic elements (19–21). Understanding how *P. aeruginosa* isolates, from clinical and environmental origin, adapt to survive in a low-nutrient medium could increase our understanding of how *P. aeruginosa* adapts in other low-nutrient environments, including sink drains, intubation tubes, and other types of hospital equipment, human tissues, and other such reservoirs where this organism is able to thrive.

Experimental evolution provides a powerful means of understanding fundamental evolutionary processes and can also be used to increase our understanding of how virulence factors in opportunistic pathogens arise or change over time (22, 23). In this study, we used an experimental evolution system to identify how six *P. aeruginosa* strains (four clinical strains and two environmental strains) adapted to a low-nutrient, static environment over the course of multiple generations. Whether adaptations to this type of environment would result in changes to phenotypes commonly associated with growth and/or pathogenicity was then explored. We also sought to determine if long-term culture in a low-nutrient environment under static conditions would result in increased or decreased fitness against a phagocytic predator, *Acanthamoeba castellanii*, which would suggest that adaptation to such environments could result in increased virulence in other organisms. This could hold true especially as the mechanisms for evading predation by amoebae may be similar to those for evading phagocytosis by macrophages (24, 25).

## MATERIALS AND METHODS

### Bacterial strains and culture maintenance

*P. aeruginosa* strains used in this study were chosen based on their colony phenotype, pyocin profile, and source of isolation. Strain details are listed in Table 1. Cells were routinely maintained on LB (Lennox) agar plates at room temperature (22°C) or grown in LB (Lennox) broth. Prior to the initiation of an experiment, a single colony would be transferred from an LB agar plate to either glass test tubes containing 5 mL of LB broth, 96-well deep plates containing 1% HL5 medium (per L: 0.5 g KH_4_PO_4_; 0.5 g Na_4_HPO_4_; 7 g yeast extract; 14 g proteose peptone, 13.5 g glucose; purchased premixed from Formedium, Catalog #HLG0102), or 96-well polystyrene plates containing 180 µL of LB broth and incubated overnight at 37°C with or without aeration as indicated. When indicated, mid-log cultures were generated from overnight liquid culture by transferring 100 µL of overnight culture to 5 mL of sterile LB broth and incubated at 37°C with aeration for 90 min.

**TABLE 1:** Strains used in the study and their pyocin profiles

Strain	Origin	Colony phenotype	S1-type	S2-type	S1S2 immunity	R-type	F-type	Reference
PA B80398	CF sputum^*a*^	Normal colony	1^*b*^	0	0	1	0	(26)
PA B80427	CF sputum	Small colony	0	1	1	1	1	(26)
PA B84725	CF sputum	Mucoid	0	0	0	1	1	(26)
PA3	CF sputum	Normal colony	0	1	0	1	0	(26)
SRP 17-047	Bathroom sink drain	Normal colony	1	0	1	1	0	(27–29)
SRP 17-055	Kitchen sink drain	Normal colony	1	0	1	1	1	(27–29)

^*a*^ CF indicates isolates taken from sputum samples from individuals with cystic fibrosis.

^*b*^ 1 indicates the presence of the pyocin gene in the genome, 0 indicates absence from the genome.

### Experimental evolution methods

Each strain was cultured in 8 mL of 1% HL5 in 6-well tissue culture plates. The plates were incubated at room temperature under static conditions. Every 2 to 3 days, the medium was removed with vacuum suction and replaced with 8 mL of fresh sterile 1% HL5 medium. The evolving populations were also passaged every 7 days by removing the supernatant, adding 4 mL of sterile 1% HL5, and scraping with a cell scraper to dislodge the adherent cells. The suspended cells were transferred to a sterile 15 mL conical tube and centrifuged at 6,976 × *g* for 15 min at room temperature. The pellets were resuspended in 1 mL of 1% HL5, and 500 µL was added to 8 mL of sterile 1% HL5 in fresh 6-well tissue culture plates. A strain collection was generated at bi-weekly time points by transferring 500 µL of culture to 500 µL of sterile LB + 40% glycerol (glycerol 20% final concentration) with subsequent storage at −80°C. At each dilution step, lineages were tested for contamination via colony PCR for pyocin biosynthesis genes. Pyocins are proteins exclusively produced by *P. aeruginosa*. The pyocin gene profiles were used to ensure that no cross-contamination occurred between the strains throughout the experimental evolution via colony PCR using published primer sequences for the specific pyocin genes (30). Three biological replicate lineages for each strain were evolved for a total of 12 weeks.

On the rare occasion that a deviation of expected pyocin profiles was observed for any given gene, the pyocin profiles were examined in the following week to determine if the trend was conserved. In all cases, the pyocin profiles returned to the expected pattern. Two strains that were originally included in the data set (not shown) were removed due to repeated loss of pyocin gene positive results, which could result from contamination or simply selection against these genes. However, we note that the final, analyzed isolates used in this study (the 12-week isolates) all matched the respective ancestral pyocin profile.

### Generation time

Overnight cultures of ancestral and evolved isolates were diluted (1:100 B83098, 17-047, 17-055, or 1:200 for B84725, PA3, and B80427) into 15 mL 1% HL5 medium in 125 mL baffled flasks. Flasks were incubated with shaking at 37°C. Every hour, the O.D._600_ was measured from 1 mL of culture and plotted in GraphPad Prism v 5.04. At time points within the exponential growth phase, samples were taken for survival analysis via serial dilution and drip plating on LB square petri dishes. Average bacterial concentrations were calculated for each of three biological replicates for each ancestral and evolved isolate.

### Cell size

Overnight cultures of ancestral and evolved isolates were generated in 5 mL of LB broth in glass test tubes with aeration. Cultures were diluted 1:100 in 1% HL5 and grown to mid-log phase (typically O.D._600_ between 0.1 and 0.15 in this minimal medium, except for strain 17-047 in which samples were taken at O.D._600_ ~0.06 at 22°C or O.D._600_ ~0.25 at 37°C—see growth curves in Fig. S1). Twenty microliters of log phase culture were transferred onto a glass microscope slide along with a drop (~20 µL) of 1% Congo red staining solution. The mixture was smeared across the slide using a coverslip, then allowed to dry for 5 min in a biosafety cabinet. The slides were imaged under a 1,000× magnification light microscope with an ocular micrometer for scale. ImageJ was used to quantify the length of cells. At least 100 cells from at least two independent experiments (*n* > 200 for each isolate/strain) each were measured.

### Biofilm biomass quantification

Biofilms were quantitated via a slightly modified version of a previously published protocol (31). Briefly, overnight cultures of ancestral and evolved lineages in replicates of four of each isolate were generated in 180 µL of LB in 96-well dilution plates without aeration. Biofilms were set up by first using a 96-well transfer apparatus to transfer inocula of overnight cultures to fresh 96-well PVC plates containing 100 µL of 1% HL5. The inoculated plates were sealed with porous adhesive film (VWR, 60941-086), loosely wrapped with aluminum foil, and then placed in a humidity chamber with sterile water at the bottom. The lid of the humidity chamber was loosely sealed to allow for air flow while also maintaining a moist environment. The plates were incubated at 37°C for 48 h or 7 days to allow for biofilm formation. Biofilms were washed two times with water, then 125 µL of 0.1% crystal violet was added to each well, and the plates were allowed to incubate at room temperature for 10 min. Afterward, the biofilms were washed four more times with water. Then 200 µL of 30% acetic acid solution was added to each well and allowed to incubate at room temperature for 15 min. A 96-well plate reader was used to quantify crystal violet in solution at an optical density of 500 nm.

### Pyocyanin and pyoverdine production

Pigment levels from *P. aeruginosa* strains were determined using protocols modified from a previously published study (32). Overnight cultures were generated in 5 mL of LB broth with aeration in glass test tubes. One hundred microliters of the overnight culture was transferred to 1 mL of LB broth in 24-well plates. The plates were sealed with porous adhesive film (VWR, 60941-086) and incubated at 37°C for 48 h under static conditions. Post-incubation, 1 mL of each sample was transferred from the 24-well plates to 1.5 mL microfuge tubes. The tubes were then centrifuged at 21,130 × *g* for 30 min to pellet the bacteria, and 100 µL of supernatant from each culture was transferred to either 96-well clear plates (VWR, #10861-562) for pyocyanin quantitation or 96-well black plates (Greiner, #655077) for pyoverdine. A 96-well plate reader was used to quantify the amount of pyocyanin (absorbance at 686 nm) or pyoverdine (fluorescence 395ex/460em).

### Motility assays

The motility protocol used in this study was modified from the motility assay described in reference 32. Overnight cultures of ancestral and evolved isolates (each *n* = 3) were grown in 5 mL of LB broth in glass test tubes with aeration. Briefly, a 2–10 µL pipette tip was dipped in the overnight culture, then was used to stab approximately halfway deep into the motility agar (10 mL of 0.3% LB agar in 6-well plates). The inoculated plates were incubated at room temperature for 24 h. The diameter of the spread was measured using a metric ruler to quantify swimming distance. Isolates from each replicate lineage were tested at 12 weeks. For those that decreased, isolates were then tested at 2, 3, 5, and 7 weeks, but only until the time when motility appeared to be significantly reduced.

### *Acanthamoeba castellanii* culture and maintenance

*A. castellanii* strain 30010 was obtained from the ATCC, and cells were routinely maintained at room temperature in T-75 tissue culture flasks containing 15 mL of 100% HL5 growth medium. The cells were passaged routinely when they reached confluence, approximately every 3 days, using a cell scraper to dislodge the cells from the bottom of the flask, then removing all but 1 mL of culture from the flask and replacing it with 14 mL of sterile 100% HL5. Cell stock cultures were maintained up to four passages.

### Co-culture experiment with *Acanthamoeba castellanii*

After reaching confluence, approximately 6 × 10^3^ *A. castellanii* cells, based on hemocytometer counts, were transferred to wells of 6-well tissue culture plates along with 4 mL of 100% HL5 medium. The cells were incubated at room temperature for 1 h to allow the cells time to anneal to the surface of the wells in the 6-well tissue culture plates. The 4 mL of 100% HL5 was then removed, and the wells were washed two times with sterile 1× PBS, then 8 mL of 1% HL5 was added to the wells. Overnight cultures of ancestral *P. aeruginosa* and one of the evolved lineages of each *P. aeruginosa* strain were generated in triplicate in 5 mL of LB broth. Mid-log cultures were generated as previously described above until an O.D._600_ between 0.8 and 1.0 was reached for all strains. *P. aeruginosa* cells were added to the 6-well tissue culture plates in a ratio of 1 *P*. *aeruginosa* cell to 10 *A*. *castellanii* cells in three biological replicates per *P. aeruginosa* isolate.

The plates were incubated at room temperature for 16 days. Every 3 days, the medium was replaced by using vacuum suction and replaced with 8 mL of sterile 1% HL5. *A. castellanii* cells were enumerated on days 0, 3, 6, 8, 11, 13, and 16 via direct cell count at 400× magnification. *P. aeruginosa* cells were enumerated on day 16 via serial dilution and plating on LB agar plates.

### Genome sequencing

One milliliter of each ancestral and one of the evolved lineages for B80398, B80427, 17-047, and 17-055 was grown in LB medium, and genomic DNA was isolated using a commercial kit (Promega Wizard Genomic DNA Purification Kit) according to the manufacturer’s instructions. DNA was shipped on dry ice to Novogene for genome sequencing. A total amount of 0.2 µg DNA per sample was used as input material for the DNA library preparations. Briefly, the genomic DNA sample was fragmented by sonication to a size of 350 bp. Then, DNA fragments were end-polished, A-tailed, and ligated with the full-length adapter for Illumina sequencing, followed by further size selection and PCR amplification. After PCR products were purified by the AMPure XP system (Beverly, USA). Subsequently, library quality was assessed on the Agilent 5400 system (Agilent, USA) and quantified by QPCR (1.5 nM). The qualified libraries were pooled and sequenced on Illumina NovaSeq platforms with a PE150 strategy at ~100× coverage, according to effective library concentration and data amount required.

### Variant calling analysis

To compare ancestral and evolved lineages and measure punctual changes related to adaptation, we performed variant calling analysis. After sequencing, raw read quality control was performed using FastQC v.0.20.0 (33) to identify adapters and then remove them using Cutadapt v4.2 (34). We then proceed to trim sequences with low-quality bases using Trimmomatic (35) with the following parameters LEADING:10 TRAILING:3 SLIDINGWINDOW:4:30 MINLEN:35. Paired-end high-quality reads were mapped to the reference genome, *P. aeruginosa* UCBPP-PA14, using Burrows-Wheeler Aligner v.0.7.17 (36). Post-alignment processing, including sorting, indexing, and duplicate removal, was conducted with Samtools (37) to ensure clean and accurate input data for variant analysis. Base recalibration and indel realignment were performed using GATK’s Best Practices workflow to correct systematic errors introduced during sequencing (33, 38, 39). Variants were called using GATK’s HaplotypeCaller, configured to detect both single nucleotide polymorphisms (SNPs) and indels across the entire genome. Stringent filtering criteria were applied to identify high-confidence variants, including minimum mapping quality scores, read depth thresholds, and variant quality scores. SnpEff (40) was used to identify the genes where the variants were located or the closest genes to a variant and to predict the effects and impacts of the variants on the genes and protein products.

### Statistics

Data were analyzed with one-way analysis of variance (ANOVA) with Tukey’s or Dunnett’s post-test or with *t*-tests as described below in GraphPad Prism 5.0.4 as indicated. Spearman correlations were calculated in GraphPad Prism 9.0. Analysis across strains and selection performed by two-way ANOVA using JMP 18.2.2. The results of the two-way ANOVAs are provided in the supplemental material (Fig. S1).

## RESULTS

To test how a variety of *P. aeruginosa* phenotypes change in response to forced growth in a low-nutrient environment, we conducted a 12-week-long experimental evolution on six *P. aeruginosa* strains. Four of these strains were of clinical origin (PA B80398, PA B80427, PA B84725, and PA3), and two strains were of environmental origin (SRP 17-047 and SRP 17-055) as they were isolated from household sink drains. We note here that because these environmental strains came from a built environment, there is an equal chance that they recently came from a human or animal source or that they have been existing in this drain environment for many generations. These strains were chosen based on their colony phenotypes, pigment production, and pyocin profiles, and these strains represent a wide range of *P. aeruginosa* morphotypes. Four of the strains (PA B80398, PA3, SRP 17-047, and SRP 17-055) present with “normal” colony morphology. The normal colony phenotype of *P. aeruginosa* is frequently isolated from acute infections or environmental samples and is associated with more aggressive virulence factors, such as pyocyin production and T3SS, as well as increased motility and a predisposition to a planktonic lifestyle (41–43). The other two strains, PA B80427 and PA B84725, are small colonies and mucoid, respectively, both of which are often isolated from chronic infections and are not known to be isolated from environmental samples. The small colony and mucoid phenotypes commonly present with defensive virulence factors to avoid detection by the host’s immune system, such as reduction in motility and increased biofilm formation (42, 44). The mucoid phenotype also overproduces the carbohydrate polymer alginate, making itm difficult to phagocytose (45).

An experimental evolution was designed to mimic the conditions that *P. aeruginosa* would encounter in a non-host environment, such as the soil or an abiotic surface, such as a sink drain. To mimic these environments, *P. aeruginosa* was cultured over time under static conditions (to encourage niche specialization) in a low-nutrient medium (1% HL5 with glucose), which contains dilute yeast extract and peptone along with sodium and potassium buffers. This medium was chosen as *P. aeruginosa* readily utilizes glucose and amino acids as carbon sources, amino acids stimulate biofilm formation and swarming activities, and for subsequent amoebae studies (26, 46–48). Every 2 days, the medium was removed via vacuum suction. This removes most of the planktonic cells, thus selecting for cells that can attach to the surface of the wells. Every 7 days, the medium was removed, and the adherent cells were scraped from the wells and diluted back into sterile 6-well tissue culture plates containing sterile 1% HL5. This process was continued for 12 weeks, generating three biological replicate lineages of the six *P. aeruginosa* strains. Interestingly, the normal and mucoid colony strains remained the same in terms of colony morphology over the course of the experiment. However, all three replicate lineages of the small colony variant, B80427, underwent a morphological shift to a normal colony phenotype during the evolution experiment. All the starting ancestral isolates and isolates from the end of the evolution experiment (i.e., 12-week evolved isolates) were tested for a variety of phenotypes associated with growth, long-term survival, or virulence.

### Bacterial growth over time

During evolution experiments, an increase in fitness of the evolved lineage compared to its ancestor usually occurs as the organisms adapt to their conditions. One global indicator of fitness in bacteria is generation time. A decreased generation time in 1% HL5 at room temperature would indicate that the evolved isolates have an increased fitness in this environment compared to their ancestors, and thus this measurement serves as a control for the experimental evolution. However, we note that our experimental conditions did not specifically select for faster generation time as we passaged attached cells, not planktonic cells. We assessed the growth of all ancestral and representative isolates from evolved lines in 1% HL5 over time and calculated the generation times for each isolate during exponential phase growth. Both generation times and growth curves in 1% HL5 were used to assess overall growth patterns at two temperatures, 22°C which is the temperature used for the experimental evolution, and at 37°C, which is the optimal growth temperature for most *P. aeruginosa* strains. At 22°C, generation times were on the order of hours, not minutes, due to the low-nutrient conditions, as well as the temperature. Most evolved isolates from three strains, B80427, PA3, and 17-047, showed a decreased generation time compared to their associated ancestral strain. This suggests that they adapted to the conditions of the experimental evolution. Two individual isolates from the other strains, in B80398 and B84725, showed an increase in generation times, while the other two evolved isolates did not significantly change compared to their ancestral parent. One strain, 17-055, did not show a change in generation times for any of the evolved isolates.

We then assessed whether evolving in 1% HL5 for 12 weeks at room temperature also conveyed an advantage at a higher temperature due to the medium alone. We calculated the generation time for all strains grown in 1% HL5 at 37°C, generally considered the optimal temperature for *P. aeruginosa* (though we note that we did not identify the true optimal temperatures for all strains in this study). Three strains, B84725, PA3, and 17-055, have the majority of evolved isolates showing a reduced generation time, while almost all of the others are not statistically different than their respective ancestors. Comparing the two temperature studies, we also observed that *P. aeruginosa* B3098 evolved line 2 showed an increase in generation time (i.e., slower growth) at both 22° and 37°, suggesting that this isolate did not adapt to the low-nutrient conditions of the experimental evolution for some reason. Interestingly, the B84725 showed a different pattern between the two temperatures with an increased generation time at 22°C and a slightly decreased generation time at 37°C. This particular strain is the only mucoid isolate in our strain panel, and it has previously been shown that the expression of alginate biosynthesis genes is higher at 20°C than 37°C (49).

By examining the growth curves of each lineage, we observed that there were many significant differences between evolved and ancestral lineages at 22°C but not at 37°C based on repeated measures one-way ANOVAs (Fig. S1). Taken all the growth metrics together, this suggests that many of the genetic changes that occurred during the evolution served to optimize growth at room temperature, and some of these changes may have additional benefits, even at higher temperatures.

### Distribution of cell lengths

**Fig 1:**
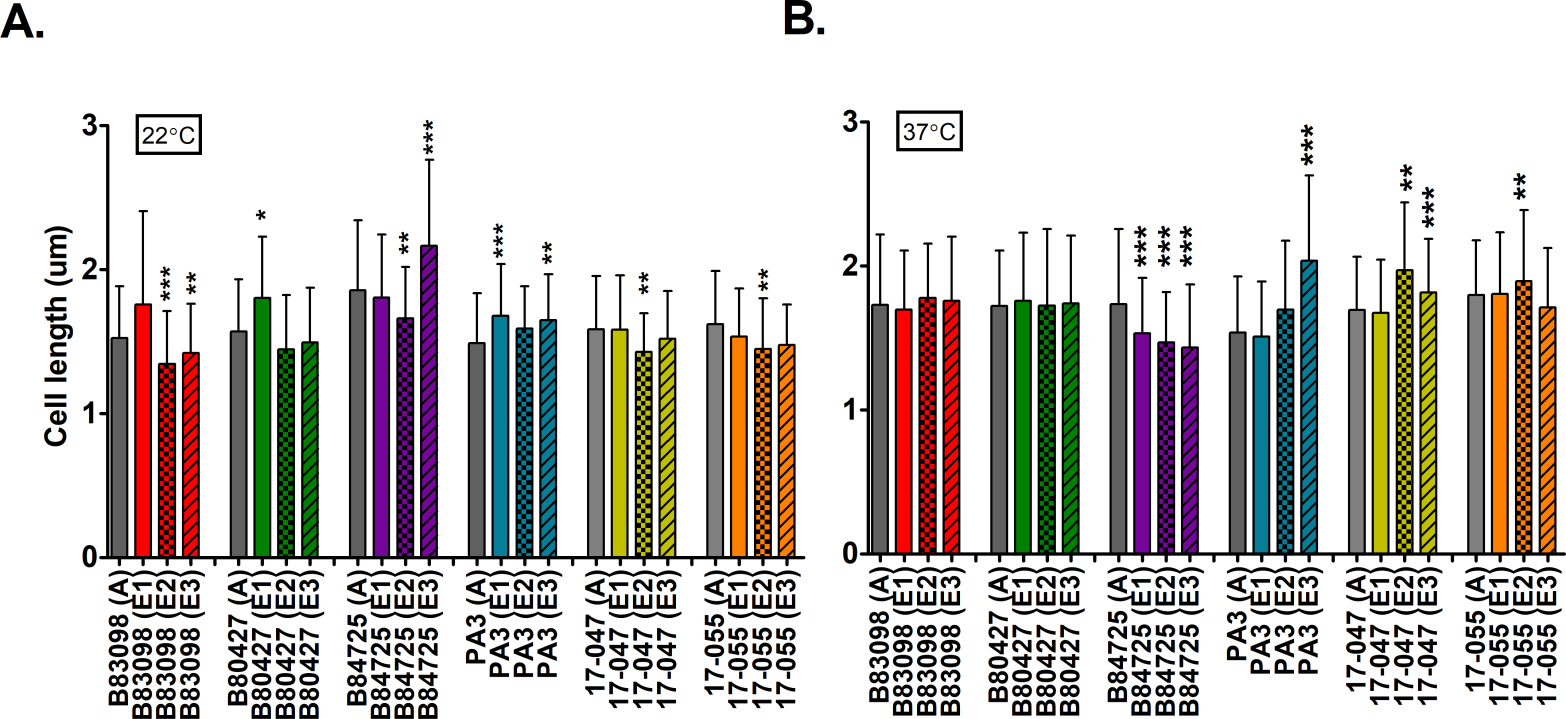
Cell size of ancestral and evolved lineages grown in 1% HL5 medium. Panels show cell sizes for strains growing at (**A**) 22°C or (**B**) 37°C. Gray bars represent average values for the ancestor strain, while colored bars represent the average of three evolved isolates. A on the x-axis indicates the ancestral isolate, and E represents evolved isolates. Data were analyzed with one-way ANOVA with Dunnett’s post-test comparing all evolved lineages to the ancestor. Error bars represent standard deviation. *P < 0.05, **P < 0.01, ***P < 0.001.

Cell size is also linked to metabolic fitness as an increased surface volume ratio results in amplified nutrient diffusion into the cell, resulting in increased metabolic efficiency, especially in low-nutrient conditions (50, 51). We hypothesized that an overall decrease in cell size would be observed in the evolved isolates, as the low-nutrient concentration of the growth medium would encourage a smaller surface area to volume ratio. Many of the evolved isolates appeared to have reduced cell length compared to the ancestor at 22°C (Fig. 1). A few evolved isolates that increased in cell length at this temperature, including B80427 evolved lineage 1 (E1) and B84725 evolved lineage 3 (E3). Curiously, the evolved lineages of PA3 all increased in size compared to the ancestor; however, this ancestral strain was relatively small to begin with, which might imply that PA3 was already adapted to lower nutrient conditions. Analysis across all strains confirms that there is a strong signal of among-strain variation (*P* < 0.0001) and strain by experimental evolution variation (*P* < 0.005), but no statistically detectable direct effect of experimental evolution (*P* > 0.5). At 37°C, most of the differences in cell length were not observed. However, all evolved lineages of B84725 showed a decrease in cell length, while a few evolved isolates, PA3 E3, 17-047 E2 and E3, 17-055 E2, had a modest increase in cell length. Our cell size data overall suggests that a small cell size was not strongly selected for, but that individual lineages had slight increases or decreases in cell length. Analysis across all strains confirms that there are strong signals of among-strain variation (*P* < 0.0001) and strain by experimental evolution variation (*P* < 0.001), but no statistically detectable direct effect of experimental evolution (*P* > 0.5). This data is confirmed when aggregating the data from the evolved lineages as well (Fig. S2).

### Biofilm formation

Another *P. aeruginosa* phenotype assessed was biofilm formation. Based on the design of our experimental evolution, we hypothesized that the evolved lineages would be superior biofilm formers compared to their ancestors because our experimental evolution design strongly selected for cells that adhered to the tissue culture surface. We assessed biofilm formation with crystal violet assays, which provide a broad quantitative means to assess the volume of biofilm biomass produced.

**Fig 2:**
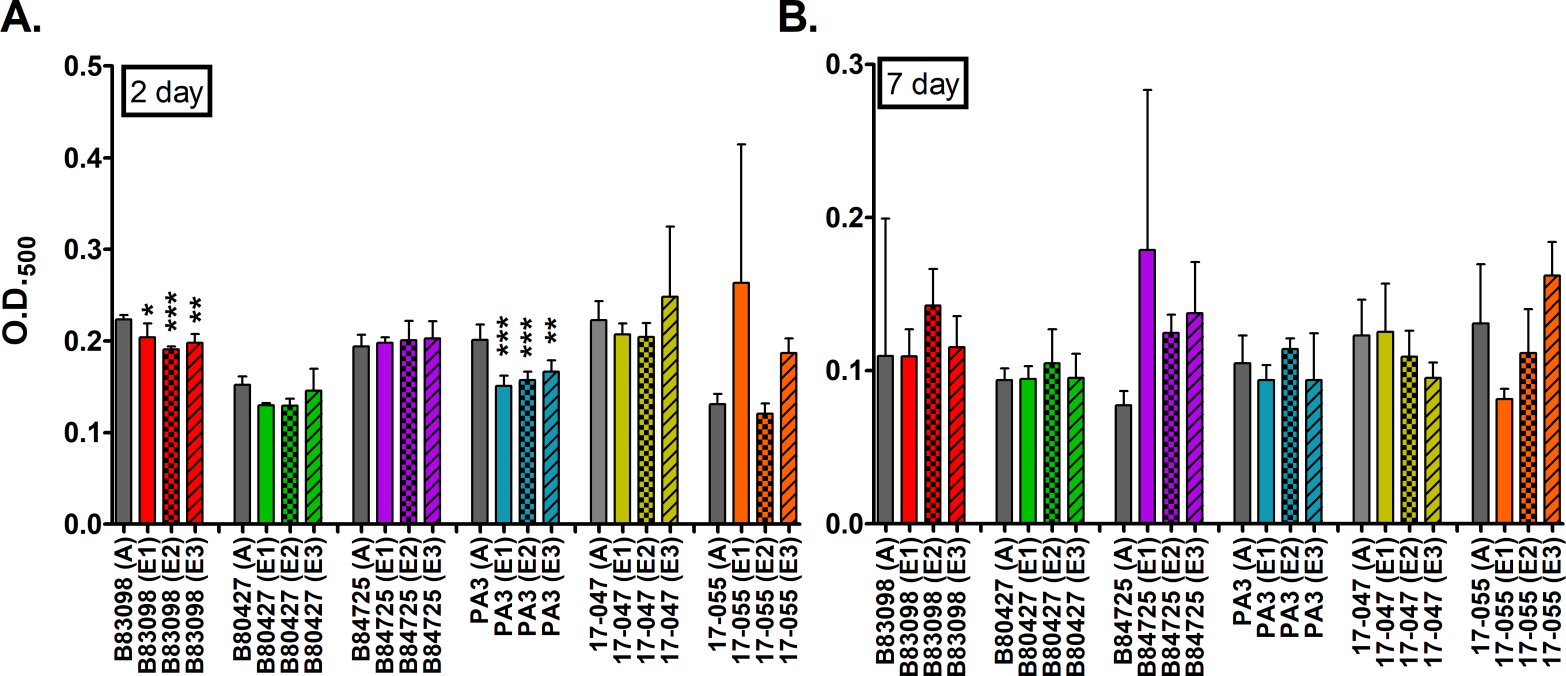
Biofilm formation of ancestral and evolved isolates in 1% HL5. Panels show crystal violet retention after (**A**) 2 days or (**B**) 7 days of incubation. Note the different y-axis scales. Gray bars represent ancestors, and colored bars represent evolved isolates. Each bar represents the average of three biological replicates for each isolate. A on the x-axis indicates the ancestral isolate, and E represents evolved isolates. Data were analyzed with *t*-tests between the ancestor and evolved lineages. Error bars represent standard deviation. *P < 0.05, **P < 0.01, ***P < 0.001.

Surprisingly, only two strains, PA B80398 and PA3, exhibited significantly different biofilm densities compared to their ancestor strains (Fig. 2). In both instances, the evolved lineages exhibited significantly reduced biofilm biomass compared to their ancestors, though the overall degree of loss is quite small (5%–25%). This result is consistent when considering each evolved lineage separately, as all lineages showed reduced biofilm biomass (Fig. S3), which suggests that this change was not driven by data from a single lineage but likely represents a true shift in the phenotype for these strains. All the remaining evolved lineages tested were not significantly different from their ancestor with respect to biofilm formation (Fig. 2; Fig. S3). Analysis across all strains and replicates confirms the complex results. There is, not surprisingly, a strong statistical signal between days 2 and 7 for biofilms (*P* < 0.0001) and for strain (*P* < 0.0001). There is more modest statistical support for strain by day (*P* < 0.05), strain by experimental evolution (*P* < 0.05), and strain by day by experimental evolution (*P* < 0.05) interactions. There is no evidence of a direct effect of experimental evolution (*P* > 0.5) nor experimental by day interaction (*P* > 0.1).

### Individual molecular phenotypes

**Fig 3:**
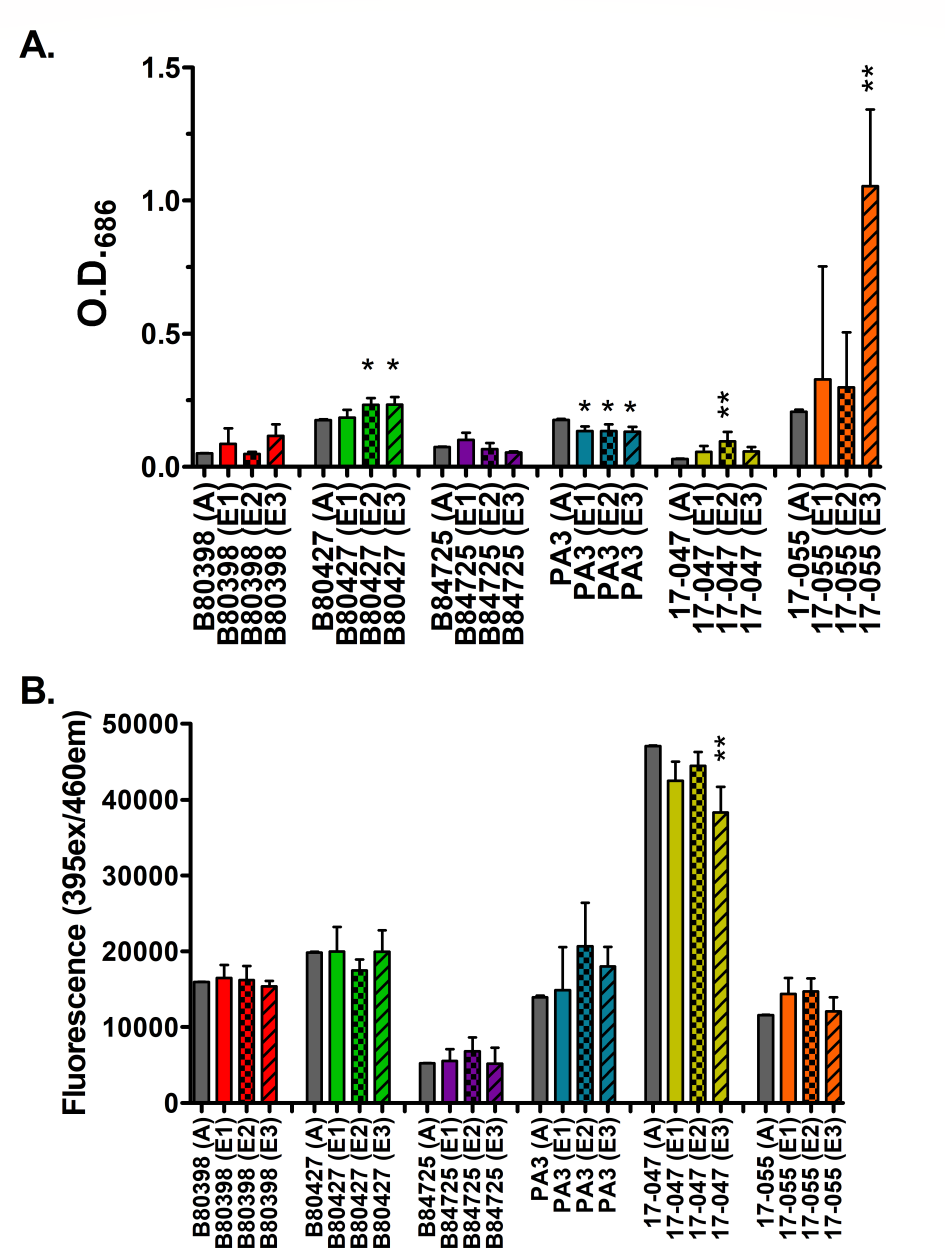
Pigment production in ancestral and evolved *P. aeruginosa* isolates. Panels show (**A**) pyocyanin or (**B**) pyoverdine production from 12-week-old evolved lineages compared to ancestors. Gray bars represent ancestors, and colored bars represent the evolved lineages. Each bar represents the average of three biological replicates for each isolate. A on the x-axis indicates the ancestral isolate, and E represents evolved isolates. Data were analyzed with *t*-tests between the ancestor and evolved lineages. Error bars represent standard deviation. *P < 0.05, **P < 0.01.

Pyocyanin is a redox-active toxin secreted by *P. aeruginosa*. It is toxic to eukaryotic cells and provides *P. aeruginosa* protection against predators, but it also serves as a secondary metabolite for *P. aeruginosa* and is associated with promoting survival in low-oxygen and low-nutrient environments (52–54). We hypothesized that the evolved lineages would produce significantly more pyocyanin than their ancestors due to the low-nutrient conditions present in our experimental evolution system. Our hypothesis was supported for three of the strains: PA B80427, SRP 17-047, and SRP 17-055, of which there was a small but significant increase in pyocyanin production in PA B80427 and SRP 17-047, and a large increase in SRP 17-055 (Fig. 3). Two strains, PA B80398 and PA B84725, did not exhibit significant differences in pyocyanin production compared to their ancestors; however, two of the individual evolved lineages of PA B80398 did produce more pyocyanin than their ancestor (Fig. S4). Interestingly, all three evolved lineages of PA3 produced significantly less pyocyanin compared to their ancestor, suggesting that pyocyanin production was not selected for in this strain. The reasons for this remain unclear. Analysis across all strains and replicates confirms the complex results. We observed a strong statistical signal for a strain effect (*P* < 0.001), but very modest statistical support for an effect of experimental evolution (*P* = 0.0289, F_1,66_ = 4.99), from a planned contrast comparing the ancestral production of all strains to all of the evolved lineages. From this data, it suggests that for most of our *P. aeruginosa* strains, pyocyanin production was selected for in our experimental evolution system.

Pyoverdine is a siderophore secreted by *P. aeruginosa,* which functions to sequester iron from other cells, as well as the environment (55). The 1% HL5 used for the experimental evolution is undoubtedly low in most macronutrients. However, it likely does contain some iron as it contains yeast extract and protease peptone, but whether the concentration was sufficient for optimal *P. aeruginosa* growth is unknown. Therefore, we could not make a hypothesis on whether pyoverdine production would change over time in the evolved isolates. Pyoverdine is highly implicated in *P. aeruginosa* pathogenicity (56), so pyoverdine production was assessed using spectrophotometry in the ancestral and evolved lineages.

**Fig 4:**
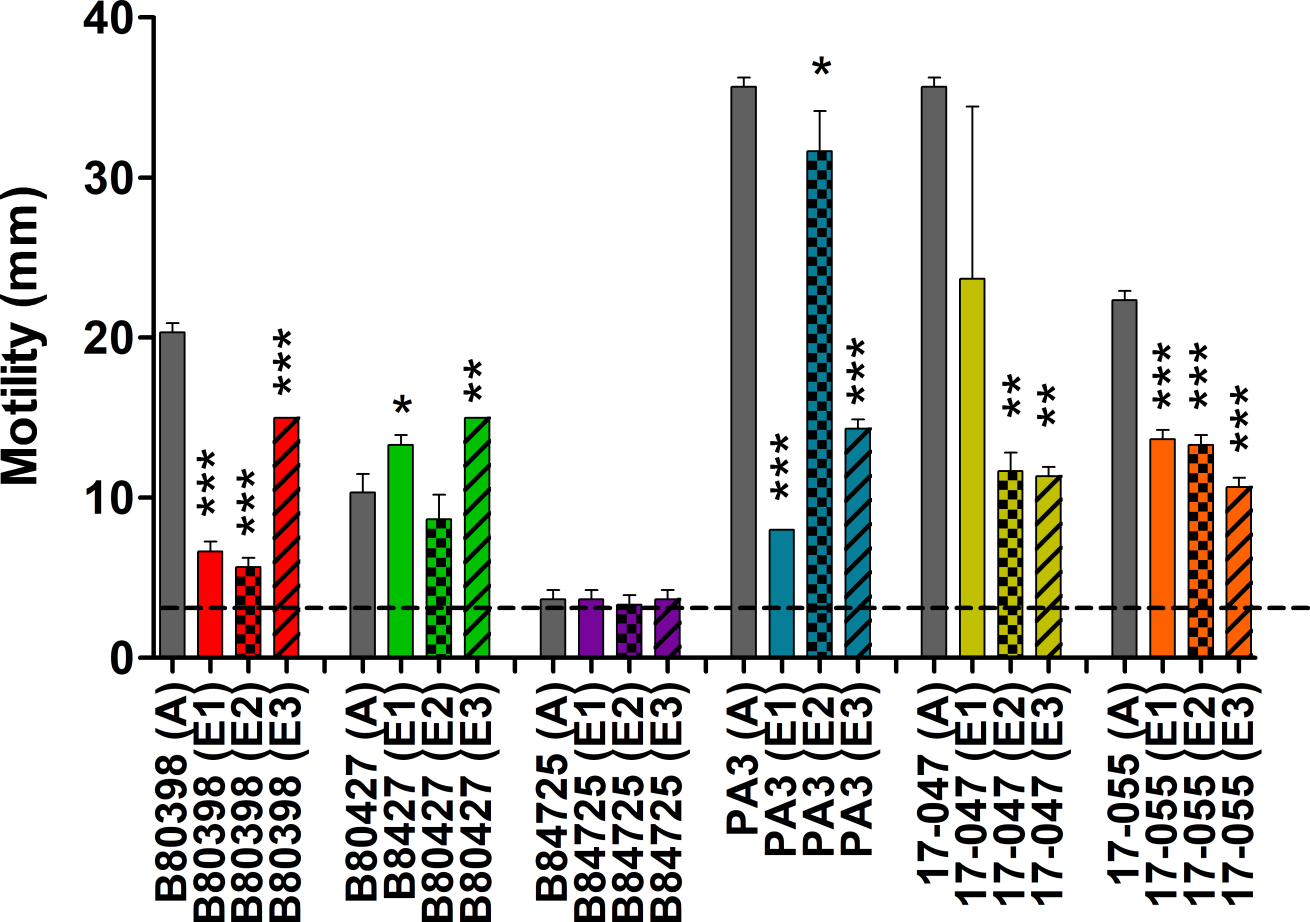
Swimming motility of ancestral and 12-week evolved isolates in 0.3% LB agar. Gray bars represent ancestors, and colored bars represent evolved isolates. Each bar represents the average of three biological replicates for each isolate. The dashed line indicates the diameter of the stab mark. A on the x-axis indicates the ancestral isolate, and E represents evolved isolates. Data were analyzed with *t*-tests between the ancestor and evolved lineages. Error bars represent standard deviation. *P < 0.05, **P < 0.01, ***P < 0.001.

Pyoverdine remained unchanged in five of the six strains tested (Fig. 4). Only one of the evolved lineages of the environmental isolate, SRP 17-047 produced significantly less pyoverdine than its ancestor, while the other two lineages of 17-047 were not significantly different (Fig. S5). One of the evolved lineages of SRP 17-055 had statistically significant higher pyoverdine production. Analysis across all strains confirms that there are strong signals of among-strain variation (*P* < 0.0001) but not for strain by experimental evolution (*P* > 0.1) or strain by experimental evolution (*P* > 0.1). This suggests that pyoverdine was likely neither selected for nor against under the conditions in our experimental evolution system.

The last molecular phenotype tested was swimming motility. We hypothesized that there would be a reduction in motility in our evolved lineages as our experimental evolution design selected against planktonic cells. Overall, we observed a strong statistical signal of effects of experimental evolution (*P* < 0.001) and strain variation (*P* < 0.01), but no statistical signal for an interaction term (*P* > 0.5). The swimming abilities of the ancestral isolates were variable from completely non-motile to highly motile (Fig. 4). There was a significant reduction in swimming motility observed for almost all the evolved lineages compared to their ancestors for B80398, PA3, 17-047, and 17-055. The other two strains, B80427 and B84725, showed very small changes in motility compared to their ancestors; however, the ancestors for these two strains were either amotile (B84725) or only weakly motile (B80427) under these conditions. Surprisingly, two of the evolved lineages, E1 and E3 of B80427, displayed small but significantly greater motility than the ancestor B80427 strain. There was no correlation between biofilm biomass and motility for all strains aggregated (Spearman r, *P* = 0.5720) or for any strain individually.

We tested the earlier isolates of the four strains that showed the greatest reduction in motility over the course of the experiment, B80398, PA3, 17-047, and 17-055, to determine when motility was lost by selecting isolates from earlier time points in the experimental evolution. Isolates from the second week of the experimental evolution were tested first, then isolates from additional weeks were tested as needed. We found that motility was lost at week 2 for 17-047, week 3 for 17-055, week 5 for PA3, and week 7 for B80398 (Fig. S5). Together, these data suggest that for strains that were highly motile at the start of the experiment, motility was selected against and loss of 49%–90% of motility between 2 and 7 weeks of culture in low-nutrient medium. The degree and timing of the loss varied for each motile strain.

### Host-associated phenotypes over the course of evolution in a low-nutrient environment

We also sought to determine if long-term adaptation to a low-nutrient environment would result in an increased or decreased ability to survive and/or kill a phagocytic predator, as this could be a strong indicator of how *P. aeruginosa* virulence can increase in low-nutrient environments. To test this, we co-cultured the ancestral isolates and one evolved lineage of each strain with the phagocytic amoeba species *A. castellanii* over time to examine the survival of both organisms. *A. castellanii,* an avid predator of *P. aeruginosa,* is a model organism for studying phagocytosis and is often used as a proxy to study mammalian macrophage engulfment (57–60). *A. castellanii* exists in two forms, trophozoites (the actively growing and metabolizing form) and cysts (dormant).

**Fig 5:**
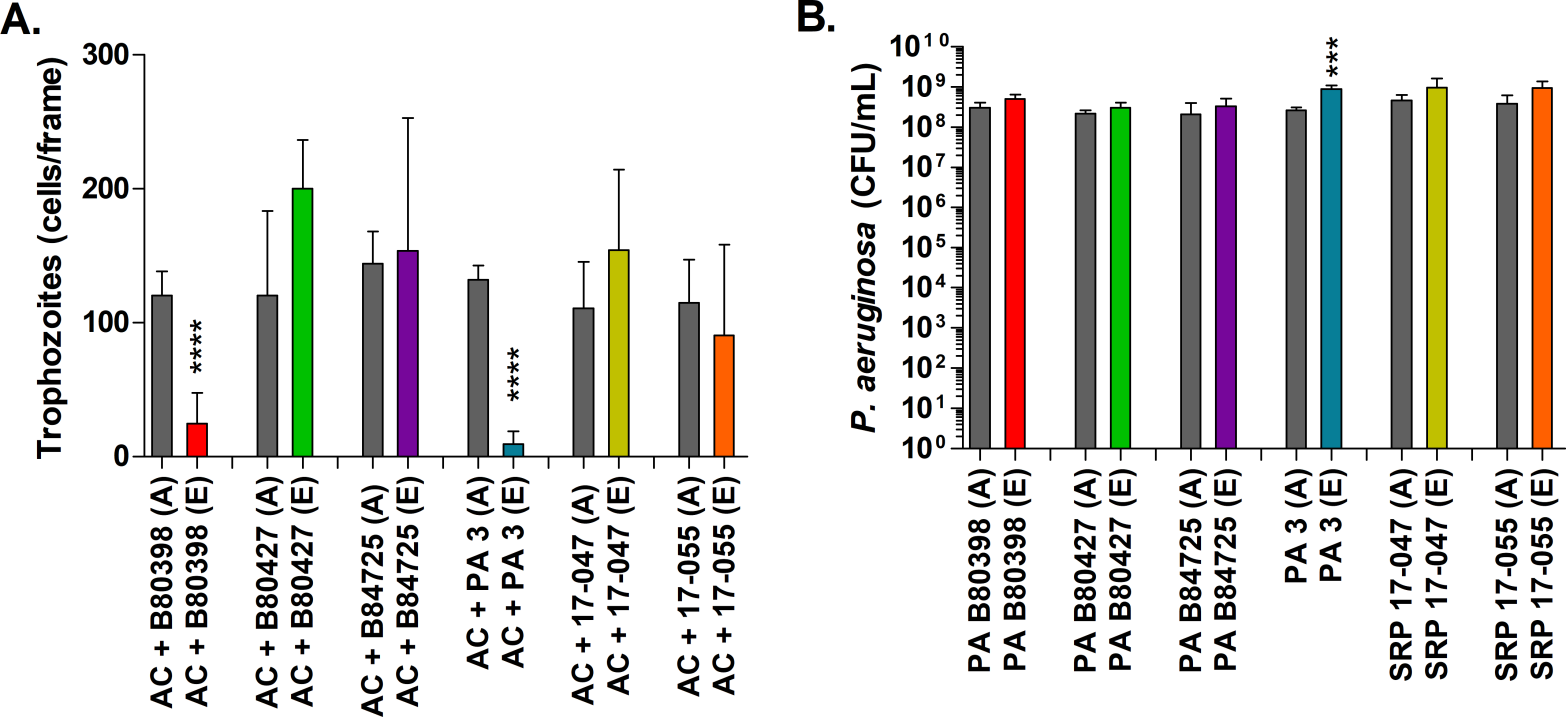
*A. castellanii* and *P. aeruginosa* survival after 16 days of co-culture. (**A**) Bars represent the average number of *A. castellanii* trophozoite counts from microscopy images. (**B**) *P. aeruginosa* viable cell counts from ancestral or a single evolved lineage in each co-culture condition (line 3 for strain SRP 17-047 and line 2 for strains B80398, B80427, B84725, PA3, and SRP 17-055) are shown. Gray bars represent ancestors (A) and colored bars represent the evolved lineage (E). Error bars represent standard deviation (*n* = 3). Data were analyzed with *t*-tests between ancestor and evolved isolates. ***P < 0.0005, ****P < 0.0001.

We examined trophozoite and *P. aeruginosa* survival for individual ancestral and evolved strains. After 16 days of co-culture, *A. castellanii* trophozoite survival was significantly reduced when co-cultured with the evolved lineage of the clinical isolates B80398 and PA3 when compared with the ancestral isolate of these strains (Fig. 5A). The remaining strains showed neither a statistical increase nor decrease in the number of trophozoites found, perhaps due to the large variability in the individual lineages for the most part. Trophozoite survival tended to be reduced over time when in co-culture with the evolved lineage compared to the ancestral isolates for most strains, but this rarely reached statistical significance for most lineages/strains (Fig. S6). The overall analysis does show statistical significance of an overall effect of experimental evolution across strains (*P* < 0.001), with strains differing (*P* < 0.01), but without statistical support for strain by experimental evolution interaction (*P* > 0.5). Even so, the co-culture dynamics of the clinical strains PA3 and B80398 over time were particularly interesting. Trophozoite levels in the presence of the ancestral strains increased dramatically over the 16 days of observation, but trophozoite levels remained very low when co-cultured with the evolved isolate of this strain (Fig. S6).

In comparison, all the *P. aeruginosa* isolates tested were able to survive in co-culture with *A. castellanii* for at least 16 days (Fig. 5B). There were no significant differences between the numbers of surviving evolved cells compared to ancestors at 16 days except for the clinical isolate, PA3, for which the number of surviving cells at 16 days was significantly greater for the evolved lineage than the ancestor. Therefore, ancestral and evolved lineages of *P. aeruginosa* were able to persist in the presence of amoebae. *P. aeruginosa* cells were only enumerated on day 16, as we were able to confirm the presence of live *P. aeruginosa* cells in the wells using bright-field microscopy at each of the other time points.

### Genomic changes in the evolved lineages compared to ancestors

To better understand the genetic mechanisms underlying the observed changes in phenotype and to better identify other potential changes, the genomes of the ancestor and one of the evolved lineages from four of the strains, B83098, B80427, 17-047, and 17-055, were sequenced and compared. The genome sizes ranged from 6.60 to 6.99 Mbp (average 6.81 Mbp), and the number of predicted genes was between 6,149 and 6,456 (average 6,318) (Table 2). The number of SNPs in coding regions between ancestor and evolved isolates varied between 212 (17-055) and 312 (B80427).

**TABLE 2:** Genome analysis summary

Strain	Ancestor (A) or evolved (line)	Mapped reads	Total reads	Mapping rate (%)	Average depth (×)	Coverage at least 1× (%)	Size (Mbp)	No. of predicted genes	No. of SNPs	No. of unique genes
B83098	A	9,766,129	11,003,760	88.75	187.1	96.11	6.89	6,410	254	66
B83098	Line 2	9,119,114	10,276,000	88.74	172.79	96.11	6.89	6,410		
B80427	A	8,921,569	9,610,204	92.83	168.34	98.18	6.60	6,149	312	47
B80427	Line 2	10,037,516	10,847,040	92.54	188.48	98.19	6.60	6,157		
17-047	A	8,676,544	9,777,748	88.74	162.52	96.65	6.83	6,253	263	62
17-047	Line 3	16,760,425	18,566,944	90.27	339.24	97.69	6.99	6,434		
17-055	A	8,866,855	10,048,604	88.24	174	97.67	6.76	6,281	W212	53
17-055	Line 2	9,461,172	10,879,064	86.97	182.51	96.65	6.92	6,456		

**Fig 6:**
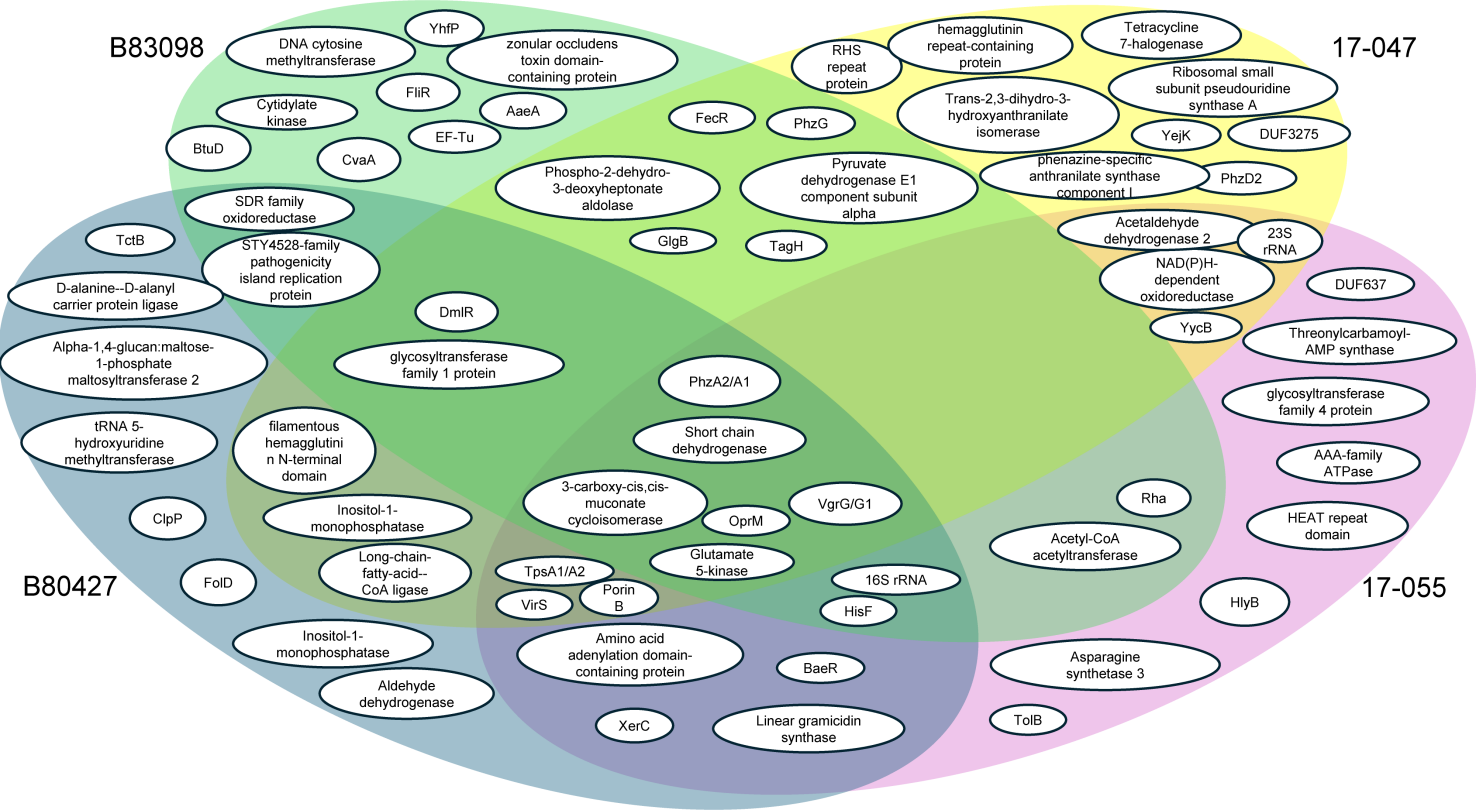
Venn diagram of single-nucleotide polymorphisms between ancestors and their respective evolved lineages after the 12-week experimental evolution. Genes with at least two SNPs in at least one of the evolved lineages are represented.

We identified coding genes that contained SNPs between ancestral and evolved lineages (Fig. 6). There were six genes or homologous gene pairs that were mutated in all four evolved lineages. They encoded: phenazine biosynthesis proteins PhzA2/PhzA1; Type VI secretion system tip proteins VgrG/VgrG1; a short-chain dehydrogenase; outer membrane protein OprM; glutamate 5-kinase (involved in the urea cycle); and 3-carboxy-cis,cis-muconate cycloisomerase (part of the protocatechuate pathway that is a central catabolic route for aromatic compounds). A number of other genes were mutated in three of the four strains. Notably, genes that encode putative virulence factors like VirS (a transcriptional regulator of virulence proteins) and TpsA/B proteins (involved in two-partner secretion of virulence proteins [61]), genes that encode histidine biosynthesis proteins. There were no genes containing SNPs that were only shared between B80398 and the two environmental strains, 17-047 and 17-055.

Additionally, SNPs that were shared between clinical strains or environmental strains were also identified. The clinical isolates B80398 and B80427 had only two unique coding regions with SNPs—an SDR oxidoreductase gene and a STY4528-family pathogenicity island replication protein. The environmental strains showed SNPs in four different genes, encoding acetylaldehyde 2, a NAD(P)H-dependent oxidoreductase, the 23S rRNA, and a gene that was annotated as YycB, an uncharacterized transporter from *Bacillus subtilis*.

In the molecular phenotype analyses, motility showed the greatest level of changes between ancestral and evolved lineages. We assessed the genomic DNA results and found that only one strain, B80398, had SNPs in a known flagella-associated protein. FliR is a core part of the FliP/FliQ/FliR complex that forms a proton motive force-dependent channel that exports flagellar proteins across the cytoplasmic membrane (62) and has also been implicated in the secretion of virulence factors (63) and in adhesion and biofilm formation (64) in other organisms. DmlR, a transcription factor implicated in carbohydrate metabolism and flagellar motility regulation, contained SNPs in three of the four strains, B80398, B80427, and 17-047 (65), though this association is only hypothetical. Evolved lineages of these strains may also have SNPs in intergenic, regulatory regions or in other types of regulatory proteins that could explain the changes in motility observed.

## DISCUSSION

**Fig 7:**
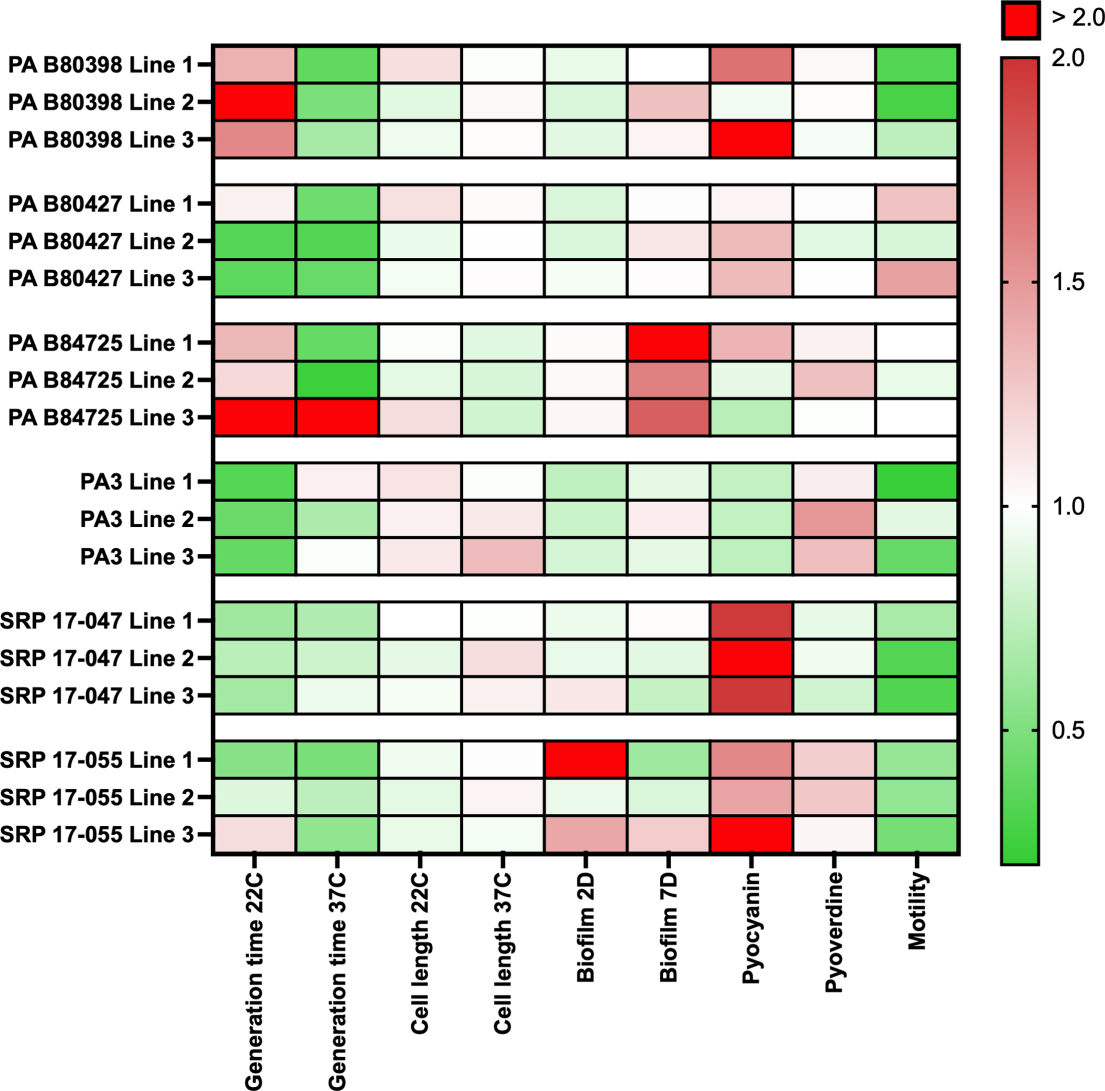
Heatmap summarizing the results of phenotypes for evolved isolates compared to the ancestors after 12 weeks of evolution in a low-nutrient medium. Red color indicates increases in values, while green indicates reductions in the values. Host-associated phenotypes were not included, as not all strains were tested. Statistics for each of these are provided in File S1.

Adaptations that occur in response to long periods without sufficient food sources could impact how bacteria grow (both in terms of how fast and how to divide their limited resources), sense and respond to their environment, their lifestyle, or how they defend themselves against predation. Culturing *P. aeruginosa* for 3 months under low-nutrient conditions resulted in alterations to multiple phenotypes associated with fitness and/or virulence (summarized in Fig. 7). Some of these observed changes in phenotype were shared amongst most of the evolved lineages, regardless of ancestral strain. These changes in phenotypes include faster generation time, reduced cell size, increased pyocyanin production, and decreased motility. Since these phenotypes were observed in most lineages, it could be inferred that these phenotypic alterations lead to increased fitness in low nutrients and thus are likely to occur in these types of environments. Two other phenotypes tested were biofilm formation and pyoverdine production, and we observed variable changes between strains and within replicate evolved lineages and within each strain. This indicates that these phenotypes were likely not as strongly selected for in our experimental evolution system (66). The lack of consistency in phenotypic changes between strains, even those isolated from similar habitats, may reflect that evolutionary changes are still occurring on route to an optimal fitness peak and/or that selecting for attached cells in these conditions may have multiple ways to manifest itself genotypically and phenotypically. Clearly, the diversity in phenotypes observed indicates a more complex route occurs than one might anticipate.

**Fig 8:**
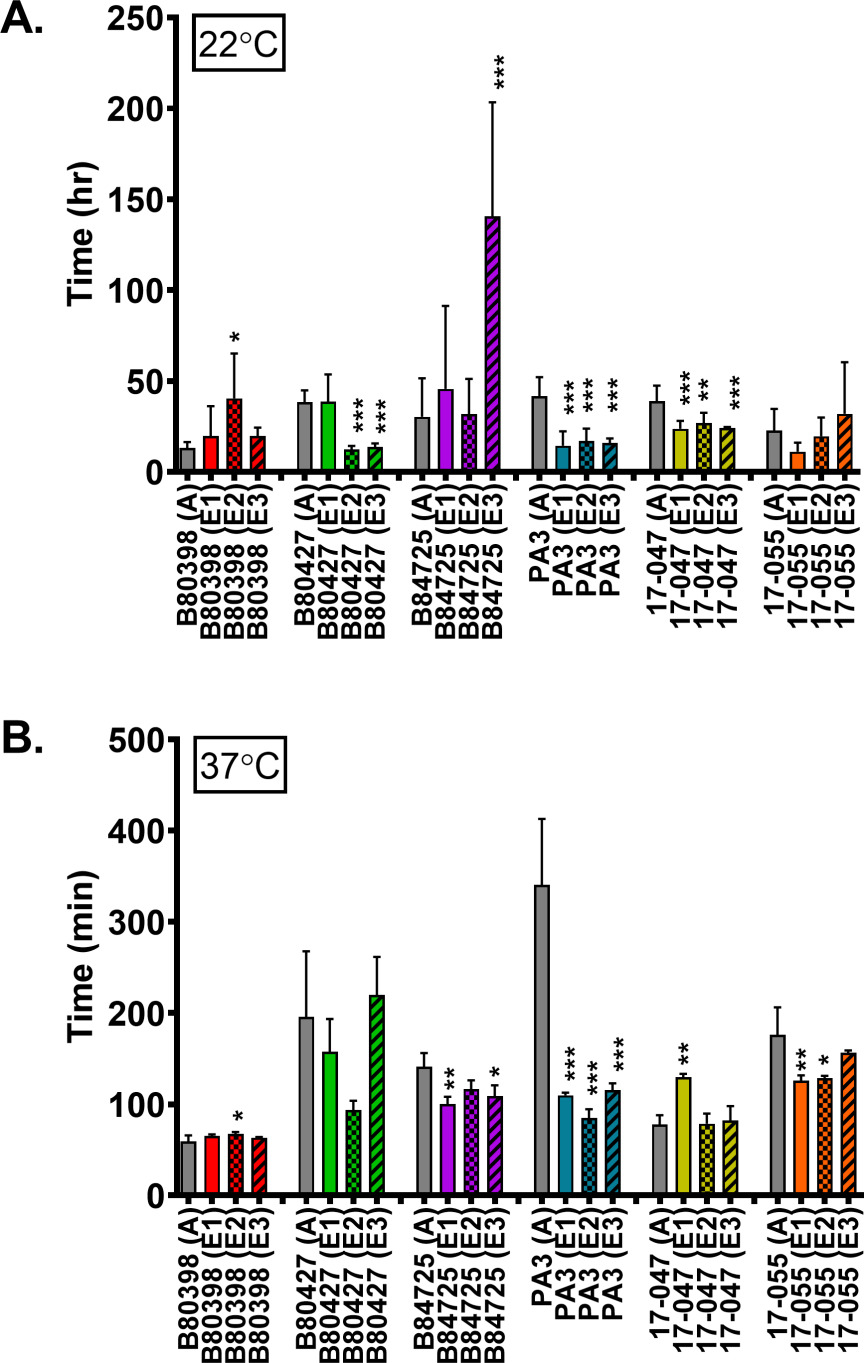
Relative generation time of ancestral and evolved *P. aeruginosa* isolates in 1% HL5. Generation times were calculated for each strain growing at 22°C (**A**) or 37°C (**B**). Gray bars represent the ancestral strains, and colored bars represent the average of three replicates of each evolved lineage. Data were analyzed with one-way ANOVA with Dunnett’s post-test comparing all evolved lineages to the ancestor. Error bars represent standard deviation. *P < 0.05, **P < 0.01, ***P < 0.001.

The environments where these particular *P. aeruginosa* strains were isolated likely had higher levels of nutrients (i.e., the clinical isolates) or fluctuating levels of nutrients (household drains). Therefore, the ancestral strains used in our experiments were likely not well adapted to constant low-nutrient conditions. Evolved lineages of three of the six strains tested exhibited significantly faster growth compared to their ancestors (at either 22°C, 37°C, or both), indicating that the evolved lineages became better adapted to the environment in which they were evolved (Fig. 8). These results are consistent with results observed in other experimental evolution systems, such as those observed in the Long-Term Evolution Experiment of *Escherichia coli*, where the doubling times of the evolved lineages reduced from 55 to 23 min in the minimal nutrient medium used in the experiment after ~50,000 generations (66). In contrast, a few of the evolved isolates (e.g., B80398 evolved line 2) exhibited significantly increased generation times compared to their ancestor (Fig. 8). Surprisingly, we did not observe any direct correlation between cell size and generation time, suggesting that cell size does not play a factor in doubling time for *P. aeruginosa* as it does for *E. coli* (50), at least not over the short-term evolution experiment described here.

Our experimental evolution system was theoretically expected to have strongly selected for increased biofilm formation. Surprisingly, evolved lineages in two of the six strains produced significantly less biofilm biomass than their ancestors, while the evolved lineages of the remaining four strains were not significantly different from their ancestors (Fig. 2). One potential explanation for this could be the low-nutrient culture medium used in the evolution experiment. Biofilms require the production and secretion of polysaccharides and extracellular proteins to form the biofilm matrix (67–69). These matrix components are undoubtedly metabolically costly to produce as the responsibility for exopolysaccharide matrix generation has been shown to be a shared responsibility within a biofilm in other species of bacteria, such as *Bacillus subtilis* (70). Thus, it is possible that the cells either acquired mutations that blocked the biosynthesis or export of some of the necessary biofilm components—perhaps choosing to use the limited carbon for growth rather than matrix production. In addition, the evolved cells could have used other methods of attachment to the plate surface, such as pili or fimbriae, to avoid the expense of secreting polysaccharides, though no SNPs were found in the coding regions of known pilin or fimbriae genes. Alternatives could include proteins like FliR, which has been shown to be involved in biofilm and adhesion (64), or be due to SNPs in transcriptional factors. Thus, there are several alternatives that could explain how *P. aeruginosa* could have attached to the surface but not initiated polysaccharide secretion. The lack of increase in biofilm formation in low-nutrient environments is similar to those observed in *P. aeruginosa* strains co-cultured over a short period with the amoebal predator, *A. castellanii,* in a minimal nutrient medium (71). In this latter study, biofilm biomass was also found to decrease in *P. aeruginosa* strains cultured in monoculture, as well as in co-culture with *A. castellanii* in minimal medium for 42 days, and gene mutations were observed in the pilin biosynthesis genes which could explain this phenomenon in their evolved strains. We did not observe similar genetic changes in most known pilin biosynthetic genes in our evolved strains.

We expected that flagellar motility would be selected against in our experimental evolution because planktonic cells were removed during every passage. A reduction in motility was observed in the evolved lineages of strains where the ancestors exhibited high motility (Fig. 4). This result is similar to results observed in *P. aeruginosa* PAO1 cultured in artificial CF medium for 120 days under aerated conditions both in monoculture and in the presence of phage competitors (72). In our study, the evolved lineages of two strains, PA B80427 and PA B84725, did not exhibit a reduction in motility, perhaps because the ancestors were poorly motile/amotile and possibly did not have sufficient loci for evolution to act upon or because there was not enough selective pressure at these loci to further reduce the motility. We did not observe a significant correlation, either negatively or positively, between biofilm formation and motility, as others have previously (73, 74). The reasons for this remain unclear, though there have been a few studies that also did not find correlations between these phenotypes in *P. aeruginosa*, particularly when *P. aeruginosa* forms aggregates (75, 76), or suggest that the flagellar dependence for biofilm formation may depend on the growth medium (77).

We also had hypothesized that pyocyanin production would be increased in the evolved populations compared to their ancestors because outside of its toxic effects on host and other prokaryotic cells, pyocyanin serves as a secondary metabolite, stabilizing the redox state of *P. aeruginosa* and can act as a final electron acceptor in the electron transport chain (42). Increased expression/production of pyocyanin should therefore increase metabolic fitness in low-nutrient environments. Pyocyanin has also been shown to play a role in pyruvate excretion and metabolism under anoxic conditions which are common in the lung environment and in biofilms (52–54). *P. aeruginosa* has been shown to be able to ferment excreted pyruvate to survive in anoxic/low-nutrient conditions for at least 18 days (53). Our pyocyanin hypothesis was supported for half of the evolved lineages when three of the six strains showed a significant increase in pyocyanin production compared to their ancestors. Because our cultures were static, it could be that increases in pyocyanin were observed for those strains that needed additional electron acceptors for their particular electron transport chain while other strains did not.

In contrast, production of pyoverdine, a siderophore, was not significantly altered in almost all evolved lineages compared to their ancestors. This result indicates that pyoverdine was not selected for or against in our experimental evolution design. This result is in opposition to results observed in previous studies which found that pyoverdine production decreased over the course of 42 days in a minimal nutrient medium (71). It is possible that the 1% HL5 used in our study contained more freely available iron and thus was not selected for or against in our experimental evolution system compared to the supplemented M9 medium used in the previous study.

In addition to identifying phenotypic changes that occurred in our evolved isolates, we also sought to determine if adaptations to a low-nutrient environment could have implications on virulence against host organisms. *A. castellanii,* a bacterivorous amoeba species, was identified as a model organism to study phagocytosis because of its commonalities with phagocytic human immune cells, such as macrophages (57–60). In our study, trophozoite survival was sometimes decreased when co-cultured with evolved isolates compared to ancestors, particularly strains B83098 and PA3, but never significantly increased when co-cultured with the ancestral isolates (Fig. 5; Fig. S6). Additionally, all the evolved lineages of *P. aeruginosa* were just as capable of surviving predation from the amoebae predator as their ancestors were (Fig. 5B). One potential reason that might underlie why *A. castellanii* trophozoites tended to survive better with the ancestors than with the evolved lineages over time could be due to the reduced motility observed in our evolved lineages. *Campylobacter jejuni, Pseudomonas fluorescens,* and *Proteus mirabilis* flagellin are recognized by *A. castellanii* to initiate phagocytosis (78, 79). Thus, if there are fewer flagella expressed in the evolved isolates, there would be less predation by *A. castellanii* resulting in starvation of the amoebae predator when in co-culture with the evolved lineages.

The genomes of the evolved and ancestral isolates showed many differences in the presence of SNPs. Whether or not these SNPs actually relate to changes in the amino acid structure of a protein or whether they influence the expression of these genes remains to be seen. However, understanding the precise mutations that occurred can shed light on the genes where natural selection may be occurring. We also note that individual isolates of evolved lines were chosen for genome sequencing, and this may have limited the resolution of the SNPs that occurred across the population. Previous studies have shown that long-term starvation, nutrient exhaustion, or feast-and-famine lifestyles can lead to increased genomic heterogeneity in bacterial populations rather than communities dominated by one phenotype produced from beneficial SNPs (80–82). Furthermore, we observed evidence of mutational parallelism among the strains after 12 weeks as was observed in reference 82, despite the feast and famine cycles somewhat mirrored in our study being much shorter than the 100 day cycles used in that study. For example, strains PA B80398 and PA 80427 showed mutations in the gene encoding the STY45280 family pathogenicity island replication protein.

In summary, a variety of *P. aeruginosa* phenotypes were observed to change over the course of long periods with limited resources, but there was a great deal of variability in how *P. aeruginosa* evolved based on individual lineage data. This may not be surprising, given the genomic plasticity within *P. aeruginosa* as a species. These results are significant as they contribute to fundamental scientific knowledge into how *P. aeruginosa* might evolve in other low-nutrient environments (such as sink drains and faucets, hospital surfaces, or freshwaters). This work could also yield insights into expected changes in *P. aeruginosa* over time in low-nutrient environments, and this could have implications for virulence properties or treatment efficacy for human infections. It could help us to understand the mechanisms of adaptation in such non-host environments (as in static cultures) and optimize sanitation protocols, especially in healthcare settings, and/or designing surfaces or materials that inhibit bacterial attachment and persistence. Lastly, this work could set the stage for future evolution experiments in which *P. aeruginosa* could be evolved with predators, such as macrophage or amoeba, to examine the effect of nutrient concentration on host–pathogen interactions.

## Data Availability

Raw sequencing data can be found in NCBI at the following BioProject ID: PRJNA1194290.

## SUPPLEMENTAL MATERIAL

File S1 aem.00975-25-s0001.pdf Statistical summaries for all two-way ANOVAs.

Fig. S1 aem.00975-25-s0002.tif Growth curves.

Fig. S2 aem.00975-25-s0003.tif Cell size.

Fig. S3 aem.00975-25-s0004.tif Biofilm biomass.

Fig. S4 aem.00975-25-s0005.tif Pigments.

Fig. S5 aem.00975-25-s0006.tif Swimming motility.

Fig. S6 aem.00975-25-s0007.tif Amoeba by strain over time.

Supplemental legends aem.00975-25-s0008.docx Legends for Fig. S1 to S6.

ASM does not own the copyrights to Supplemental Material that may be linked to, or accessed through, an article. The authors have granted ASM a non-exclusive, world-wide license to publish the Supplemental Material files. Please contact the corresponding author directly for reuse.
