# Supplementary material for: Long-term culturing of Pseudomonas aeruginosa in static, minimal nutrient medium results in increased pyocyanin production, reduced biofilm production, and loss of motility
Source: Appl Environ Microbiol. 2025 Oct 10;91(11):e00975-25. doi: 10.1128/aem.00975-25 (PMC12628827; doi:10.1128/aem.00975-25)
Supplement: File S1 — Statistical summaries for all two-way ANOVAs. [file aem.00975-25-s0001.pdf]

Supplemental summary table of two-way ANOVAs for all strains across traits

Factors	Directional Evolution	Lineage (strain)	Interaction
Biofilm	no	yes	marginal
Amoeba	yes	yes	no
Motility	yes	yes	no
Pyocyanin	no	yes	no
Pyoverdine	no	yes	yes
Size @ 22C	no	yes	yes
Size @ 37C	no	yes	yes

<u>Individual analyses:</u>

Biofilm. Strong statistical difference between day 2 and 7. Strains are different, some small interactions with evolution, but no direct effect of evolution

Source	Logworth	PValue	
Day	21.285	0.00000	
Strain	5.202	0.00001	
Strain*Day	1.734	0.01844	
Strain*Derived	1.433	0.03686	
Strain*Derived*Day	1.351	0.04460	
Derived*Day	0.545	0.28510	^
Derived	0.168	0.67911	^

Amoeba. Evolution has a strong statistical significance, with some historical (strain) effects.

Source	Logworth	PValue
Derived	3.800	0.00016
Lineage	2.249	0.00563
Lineage*Derived	0.251	0.56059

Motility. Evolution has a strong statistical significance, with some historical (strain) effects.

Source	Logworth	PValue
Derived	3.800	0.00016
Lineage	2.249	0.00563
Lineage*Derived	0.251	0.56059

Pyocyanin production. Evolution has no statistical effect, historical (strain) effects exist.

Source	Logworth	PValue
Lineage	3.200	0.00063
Derived	0.875	0.13336
Derived*Lineage	0.730	0.18601

Pyocyanin con't. Pyocyanin expression analysis across all samples (ancestors (0, 4, 8, 12, 16, & 20) and corresponding evolved (immediately following). Strong chance effects (see 21, 22, & 23).

Level	Least Sq Mean	Std Error	Mean
0	0.05096	0.0694	0.0510
1	0.08589	0.0601	0.0859
2	0.04830	0.0601	0.0483
3	0.11686	0.0601	0.1169
4	0.17630	0.0694	0.1763
5	0.18537	0.0601	0.1854
6	0.23397	0.0601	0.2340
7	0.23495	0.0601	0.2349
8	0.07459	0.0694	0.0746
9	0.10164	0.0601	0.1016
10	0.06706	0.0601	0.0671
11	0.05393	0.0601	0.0539
12	0.17735	0.0694	0.1773
13	0.13527	0.0601	0.1353
14	0.13507	0.0601	0.1351
15	0.13215	0.0601	0.1322
16	0.02924	0.0694	0.0292
17	0.05730	0.0601	0.0573
18	0.09538	0.0601	0.0954
19	0.05742	0.0601	0.0574
20	0.20755	0.0694	0.2075
21	0.32862	0.0601	0.3286
22	0.29823	0.0601	0.2982
23	1.05503	0.0601	1.0550

Pyoverdin production. Evolution has no consistent effect, historical (strain) effects exist.

Source	Logworth	PValue
Lineage	33.698	0.00000
Derived	0.392	0.40597
Lineage*Derived	0.350	0.44661

Size at 22 degrees C Evolution effect varies depending upon the lineage. No evolutionary consistent effect. Strong historical (strain) effect.

Source	Logworth	PValue
Lineage	29.733	0.00000
Lineage*Derived	2.644	0.00227
Derived	0.153	0.70283

Size at 37 degrees C. Big evolution varies depending upon the lineage. No evolutionary consistent effect. Strong historical (strain) effect.

Source	Logworth	PValue
Derived*Lineage	21.790	0.00000
Lineage	17.508	0.00000 ^
Derived	0.828	0.14857 ^

<u>Supplemental Tables Legend</u>

Supplemental tables for two-way ANOVAs for all strains across traits

Multi-way ANOVAs were conducted, with Lineage and Evolutionary state (and Day) as main effects, their interaction(s). Depending upon the analysis, the replicate lineages of the selected strains were included as nested factors (not generally shown). Outputs are effect summaries of the fixed factors. Statistical significance of one or more of the main effects was observed in every ANOVA.
