# Supplementary figures and images for: Long-term culturing of Pseudomonas aeruginosa in static, minimal nutrient medium results in increased pyocyanin production, reduced biofilm production, and loss of motility
Source: Appl Environ Microbiol. 2025 Oct 10;91(11):e00975-25. doi: 10.1128/aem.00975-25 (PMC12628827; doi:10.1128/aem.00975-25)

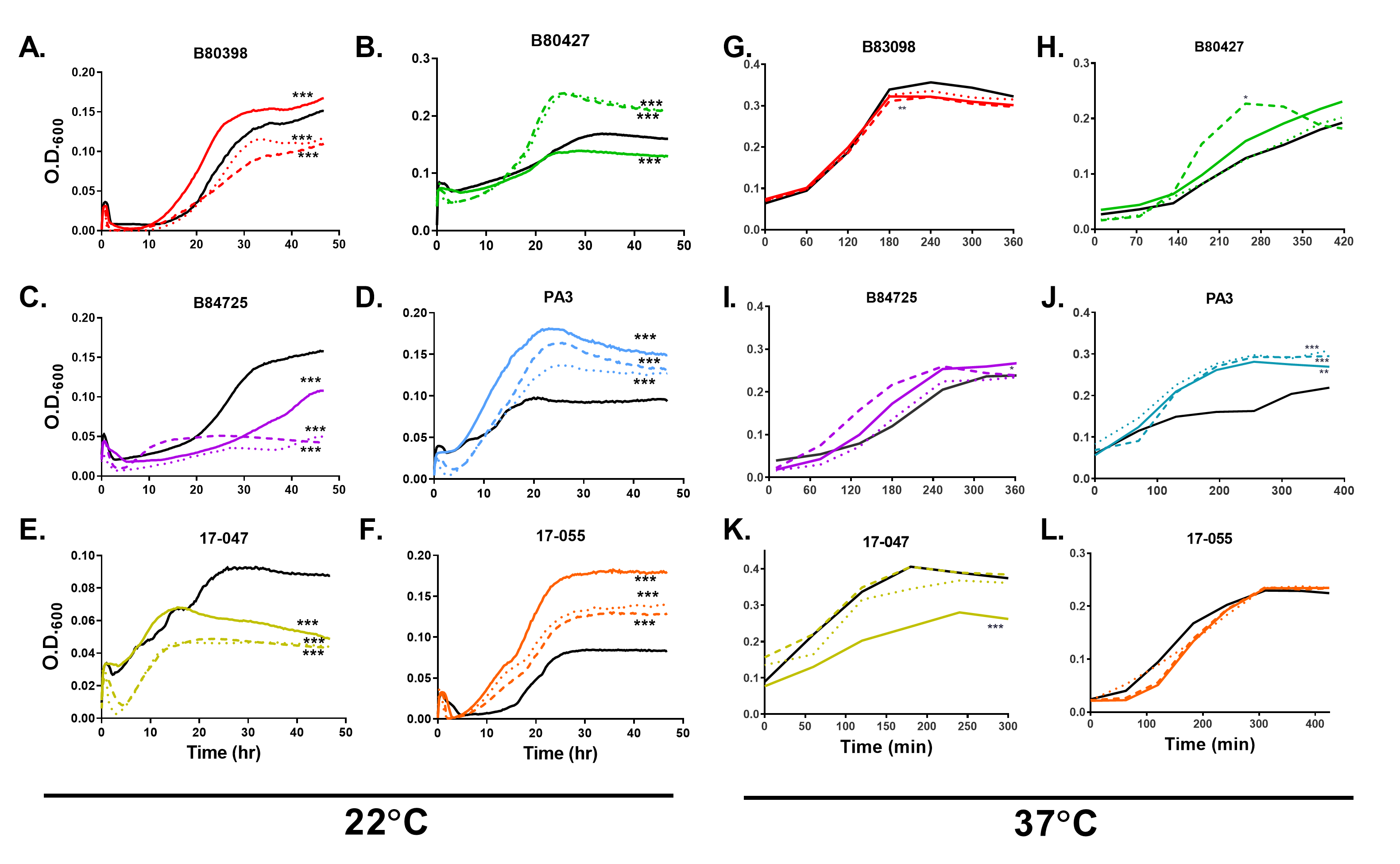

Supplement: Fig. S1 — Growth curves. [file aem.00975-25-s0002.tif]

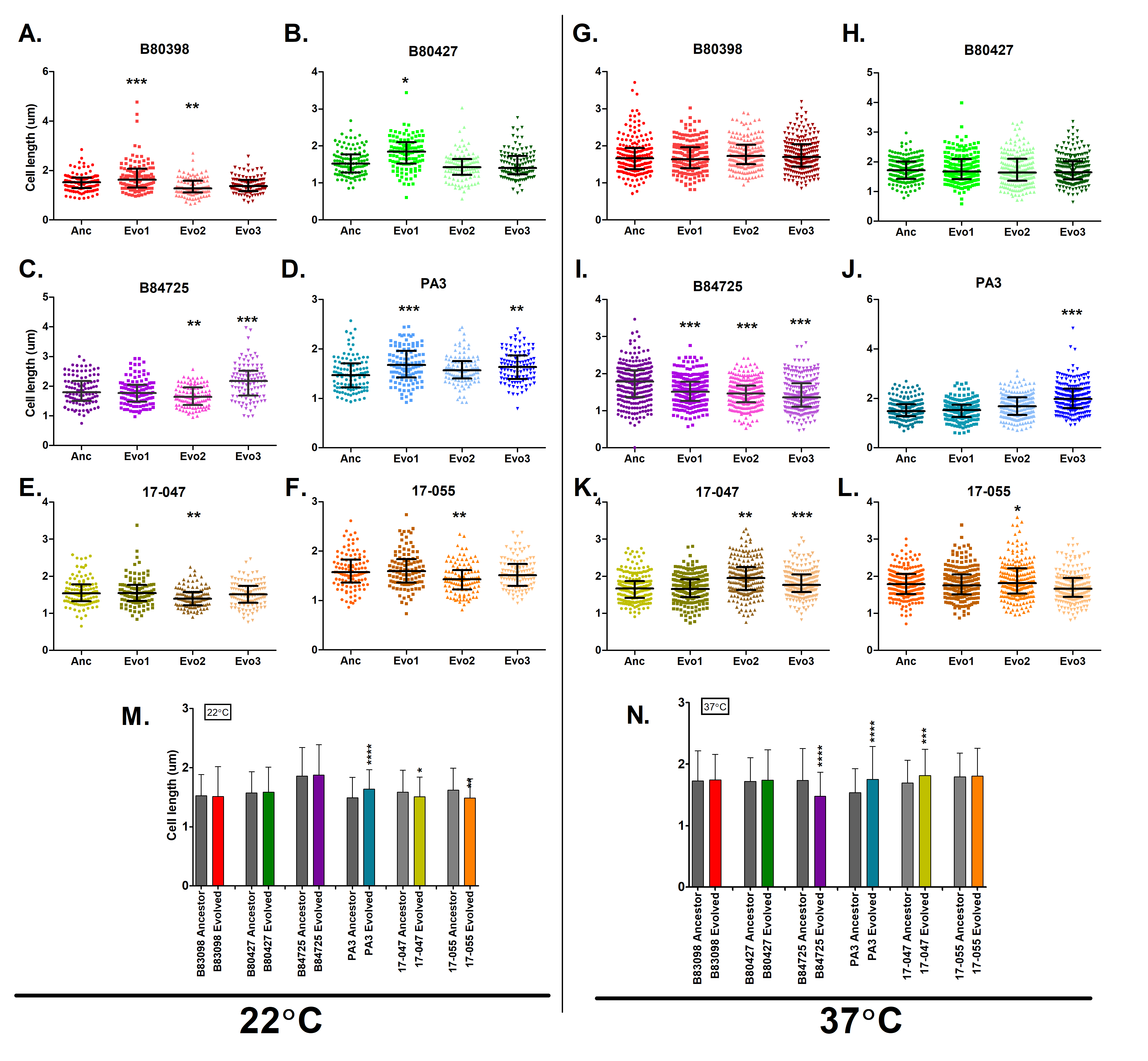

Supplement: Fig. S2 — Cell size. [file aem.00975-25-s0003.tif]

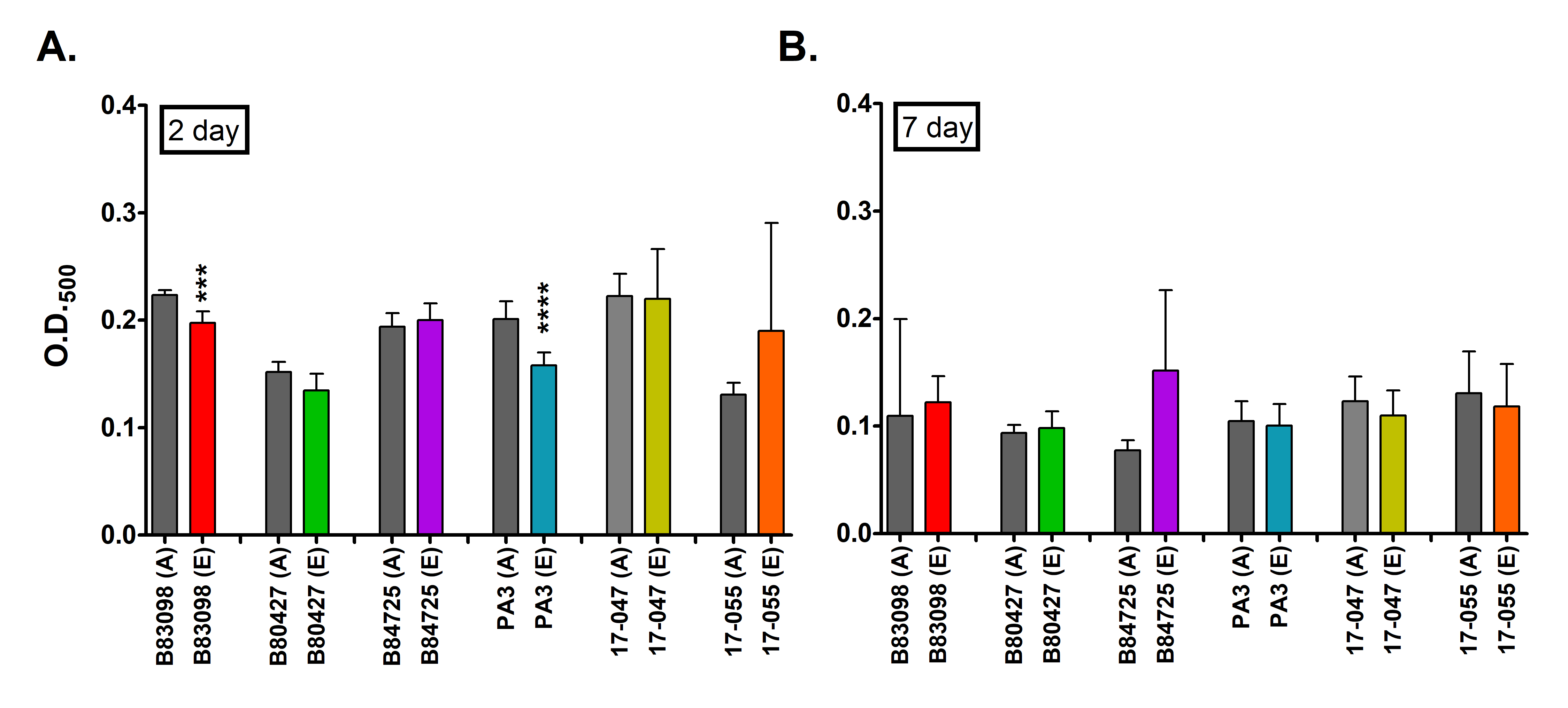

Supplement: Fig. S3 — Biofilm biomass. [file aem.00975-25-s0004.tif]

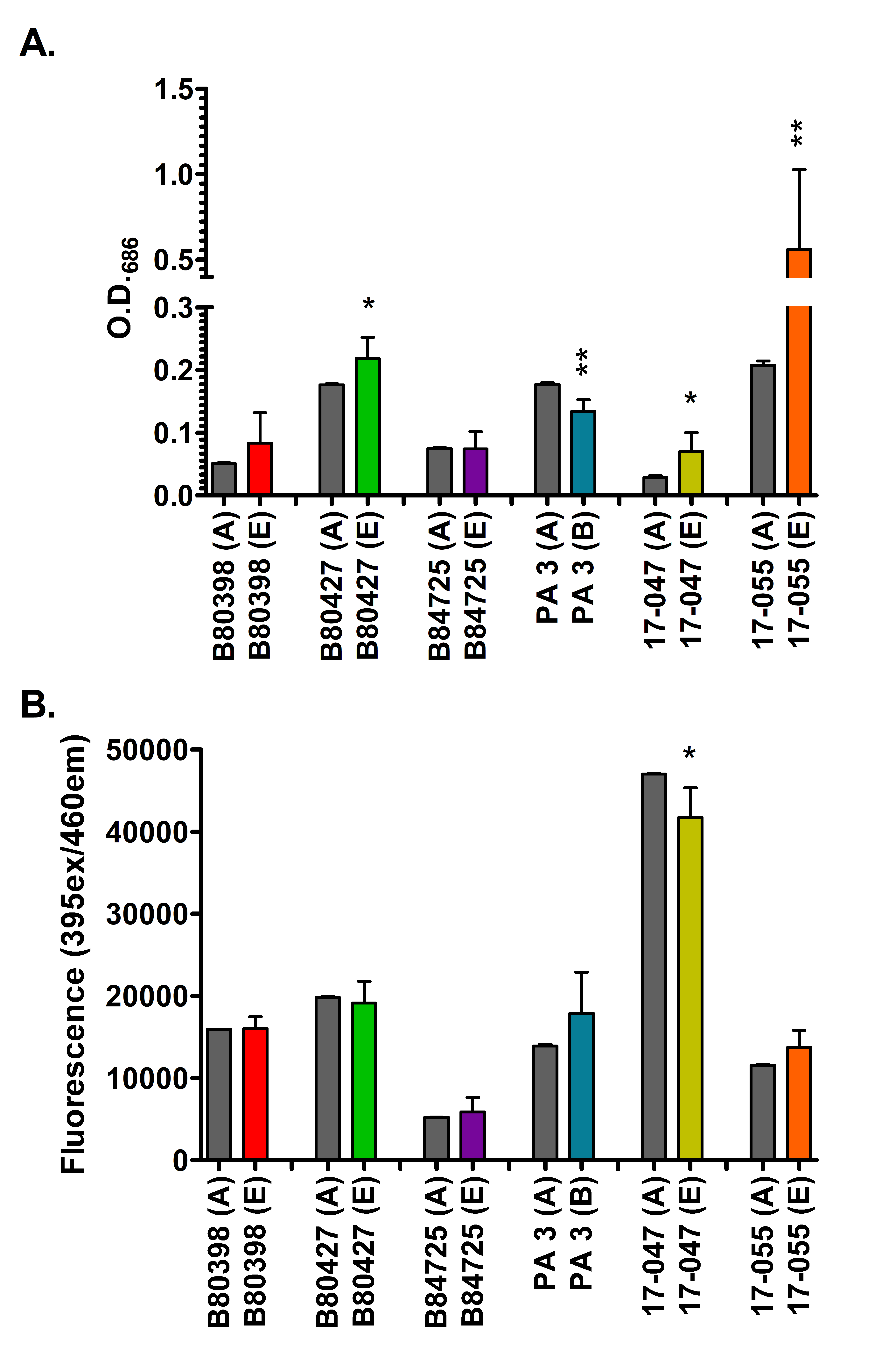

Supplement: Fig. S4 — Pigments. [file aem.00975-25-s0005.tif]

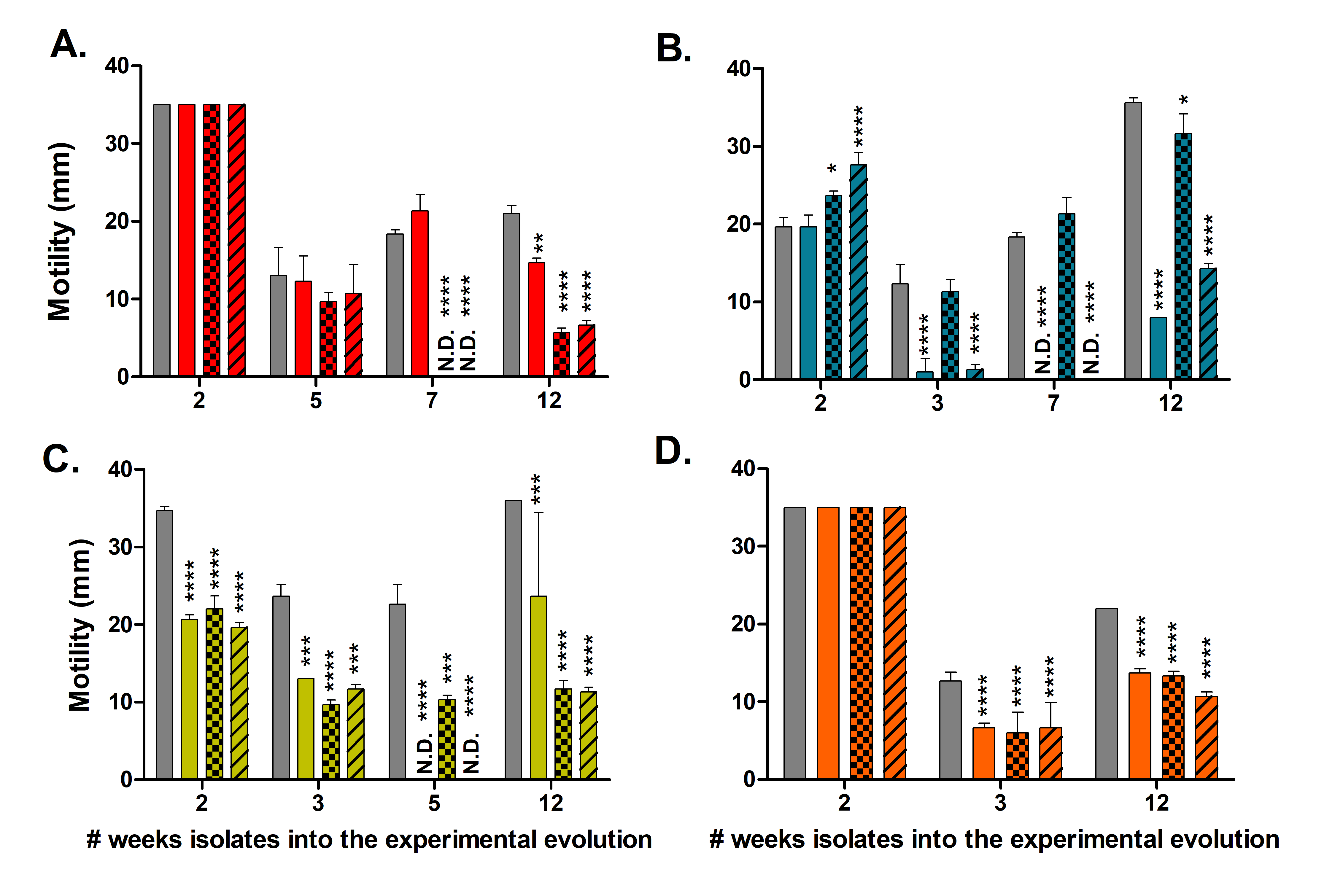

Supplement: Fig. S5 — Swimming motility. [file aem.00975-25-s0006.tif]

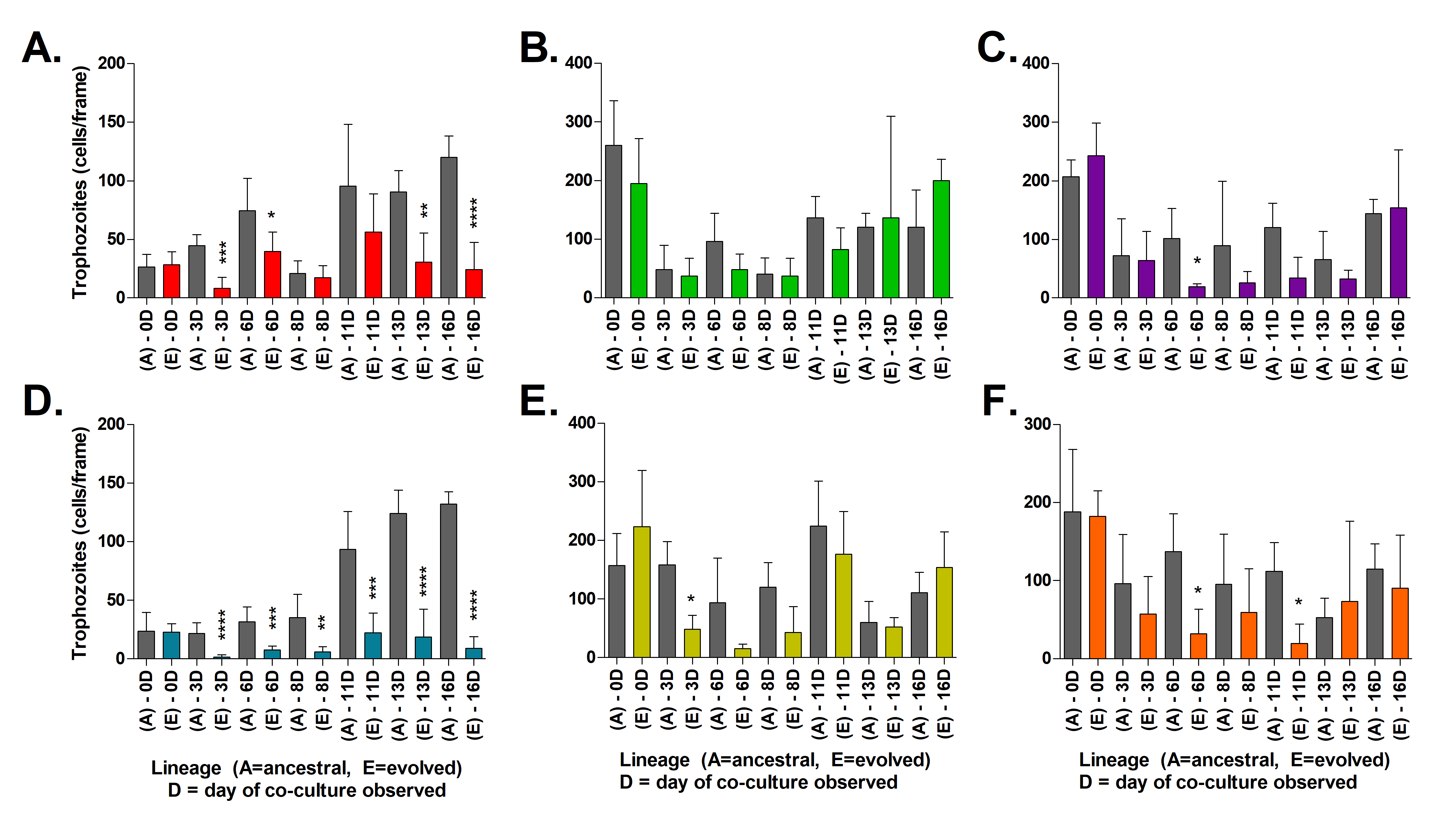

Supplement: Fig. S6 — Amoeba by strain over time. [file aem.00975-25-s0007.tif]
