# Supplementary material for: Long-term culturing of Pseudomonas aeruginosa in static, minimal nutrient medium results in increased pyocyanin production, reduced biofilm production, and loss of motility
Source: Appl Environ Microbiol. 2025 Oct 10;91(11):e00975-25. doi: 10.1128/aem.00975-25 (PMC12628827; doi:10.1128/aem.00975-25)
Supplement: Supplemental legends — Legends for Fig. S1 to S6. [file aem.00975-25-s0008.docx]

**Cecil *et al.*** “**Long-term Culturing of *Pseudomonas aeruginosa* in Static, Minimal Nutrient Medium Results in Increased Pyocyanin Production, Reduced Biofilm Production, and Loss of Motility”**

**Supplemental figure legends:**

**Figure S1.** Growth curves of the evolved lineages and their ancestors over time in 1% HL5 at room temperature (panels A-F) or 37°C (panels G-L). Lines represent the mean of 3 biological replicates. Error bars are not shown for image clarity. Data analyzed with repeat measures one-way ANOVA with Dunnett’s post-test comparing evolved lineages to their ancestor. * p<0.05, ** p<0.01, *** p<0.001.

**Figure S2**. Cell size of individual evolved lineages compared to their ancestor are shown with each cell measure represented by a dot. Cells grown at room temperature (panels A-F) or 37° (panels G-L) to exponential phase were measured. Panels M and N show aggregated data for ancestral (A) and evolved (E) isolates. Horizontal bars for each strain represent the median cell size of 100-600+ cells and error bars represent the interquartile range. Data were analyzed with one-way ANOVA with Dunnett’s post-test comparing each evolved line to its ancestor. * p<0.05, ** p<0.01, *** p<0.001, **** p<0.0001.

**Figure S3.** Biofilm biomass of individual evolved lineages compared to their ancestor after 3-month experimental evolution. Data for evolved lineages was aggregated to assess global patterns of evolution. Error bars represent standard deviation from four independent colonies from each lineage. Data analyzed with one-way ANOVA with Dunnett’s posttest comparing each evolved line to its ancestor. *** p<0.001, **** p<0.0001.

**Figure S4.** Aggregate data for evolved *P. aeruginosa* lineages for pyocyanin (panel A) or pyoverdine (panel B) production of individual isolates compared to their ancestor after the 12-week experimental evolution. Error bars represent standard deviation from three independent colonies from each lineage. Data analyzed with one-way ANOVA with Dunnett’s post-test comparing each evolved line to its ancestor. * p<0.05, ** p<0.01.

**Figure S5.** Swimming motility of individual isolates compared to their ancestor for isolates frozen at various points during the experimental evolution. Week 2 isolates were first tested, then week 3, 5, and/or 7 as needed until the loss of motility was observed. Error bars represent standard deviation from three independent colonies from each lineage. 35 mm was the largest measurable size in this analysis. N.D. indicates non-detectable growth past the stab line. Data analyzed with two-way ANOVA with Bonferroni post-test comparing each evolved line to its ancestor across time points. Error bars indicate standard deviation. * p<0.05, ** p<0.01, *** p<0.001, **** p<0.0001.

**Figure S6.** Competition assay between the ancestor and evolved lineages (line 3 for strain SRP 17-047 and line 2 for strains B80398, B80427, B84725, PA3, and SRP 17-055) of *P. aeruginosa* against *A. castellanii* at all timepoints tested. Grey bars with or colored bars represent the number of trophozoites observed when co-cultured with each ancestor (A in the x-axis) or evolved lineage (E in the x-axis) over the course of 16 days (D) Panels represent each strain with (A) B80398, (B) B80427, (C) B84725, (D) PA3, (E) 17-047, and (F) 17-055 measured at 0-, 3-, 6-, 8-, 11-, 13-, and 16 days of co-culture. Error bars represent standard deviation from three independent colonies from each lineage. Data analyzed with t-tests between ancestor and evolved lineage at each timepoint. * p<0.05, ** p<0.01, *** p<0.001, **** p<0.0001.
